# Supplementary material for: Mechanical properties of the cuticles of three cockroach species that differ in their wind-evoked escape behavior
Source: PeerJ. 2014 Jul 31;2:e501. doi: 10.7717/peerj.501 (PMC4121590; doi:10.7717/peerj.501)
Supplement: Supplemental Information 1 — Photos of cuticles and measurements. [file peerj-02-501-s001.pdf]

Calibration slides
smallest division = 0.01 µm

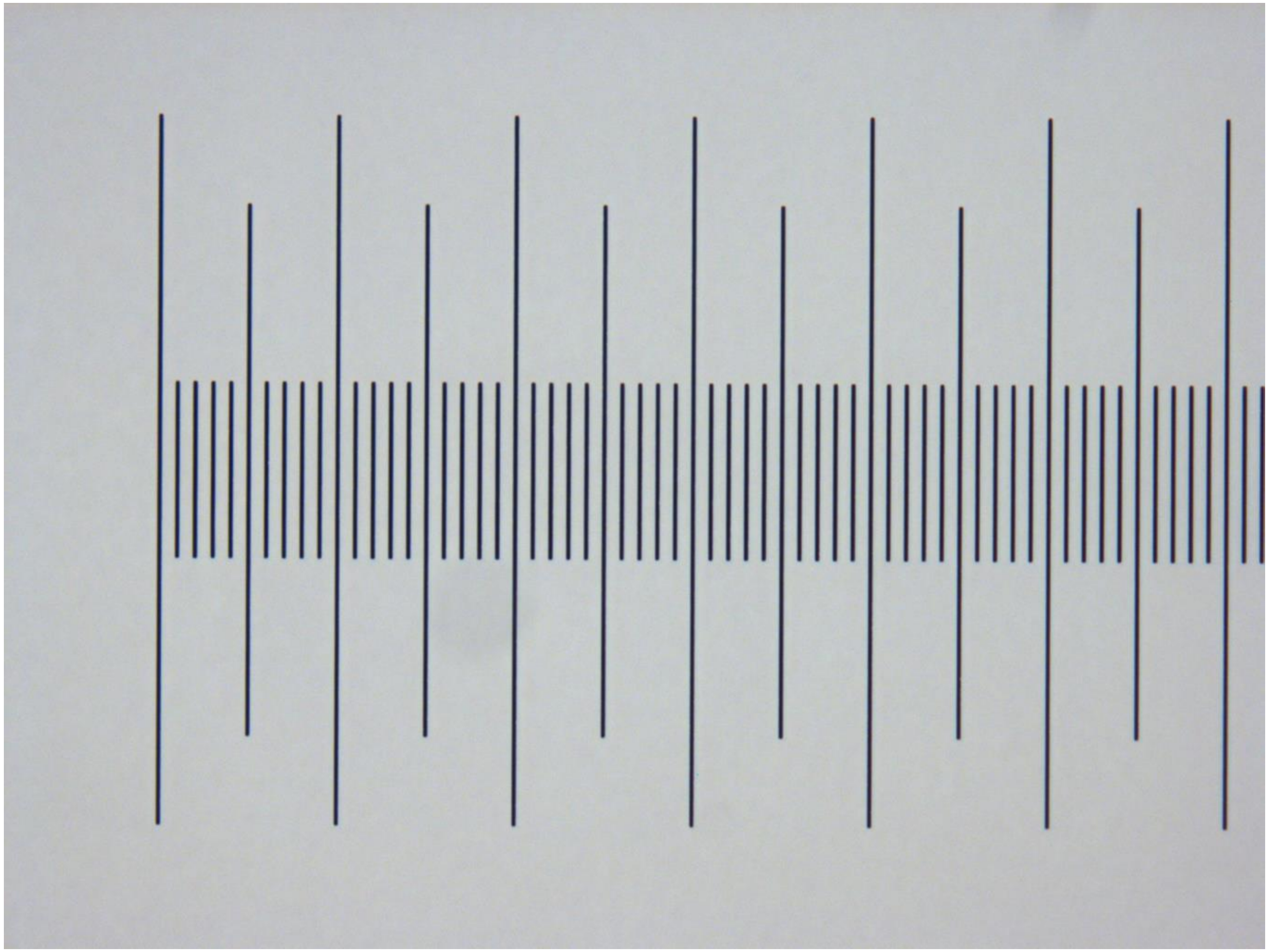

10x magnification

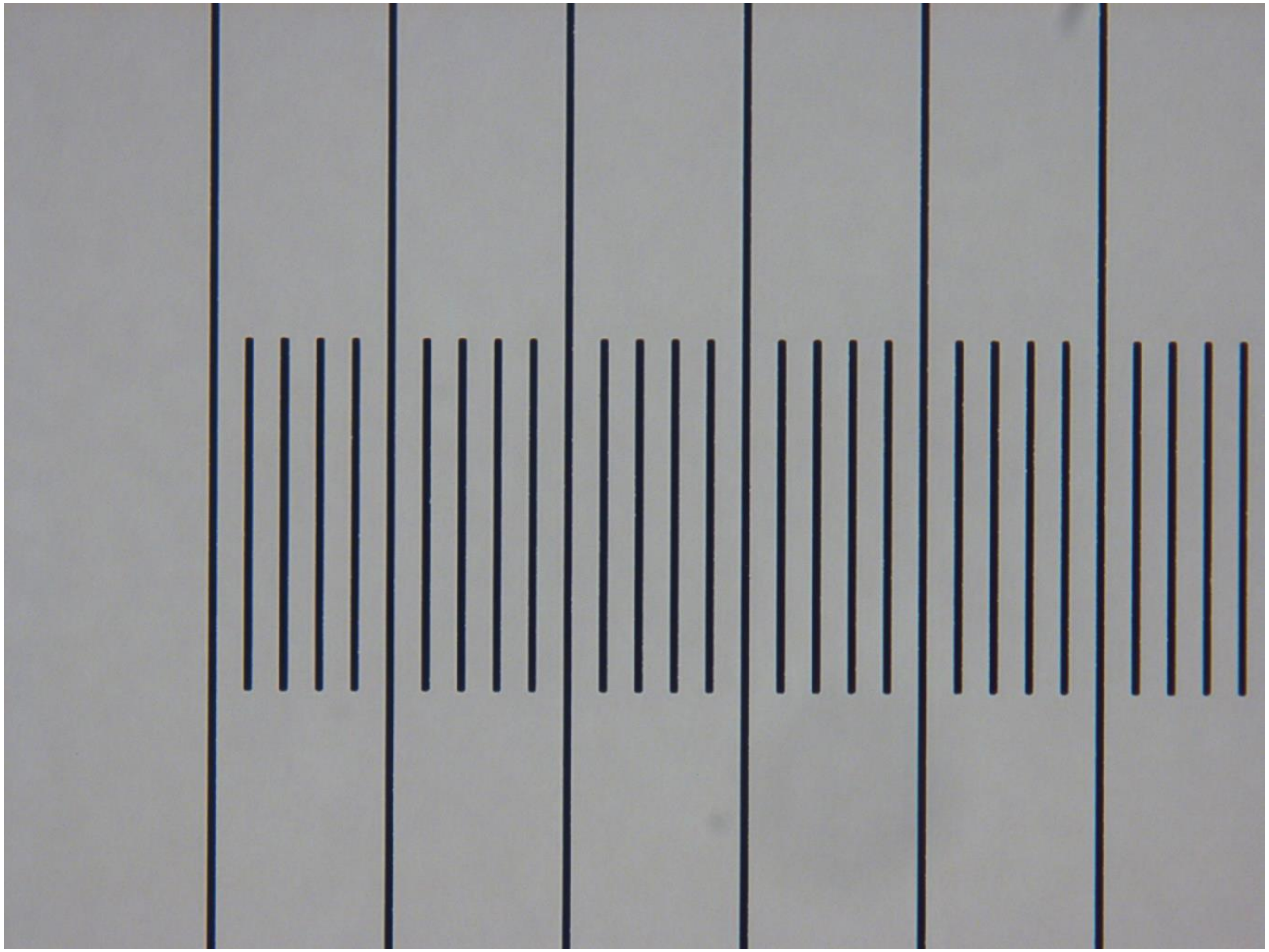

20x magnification

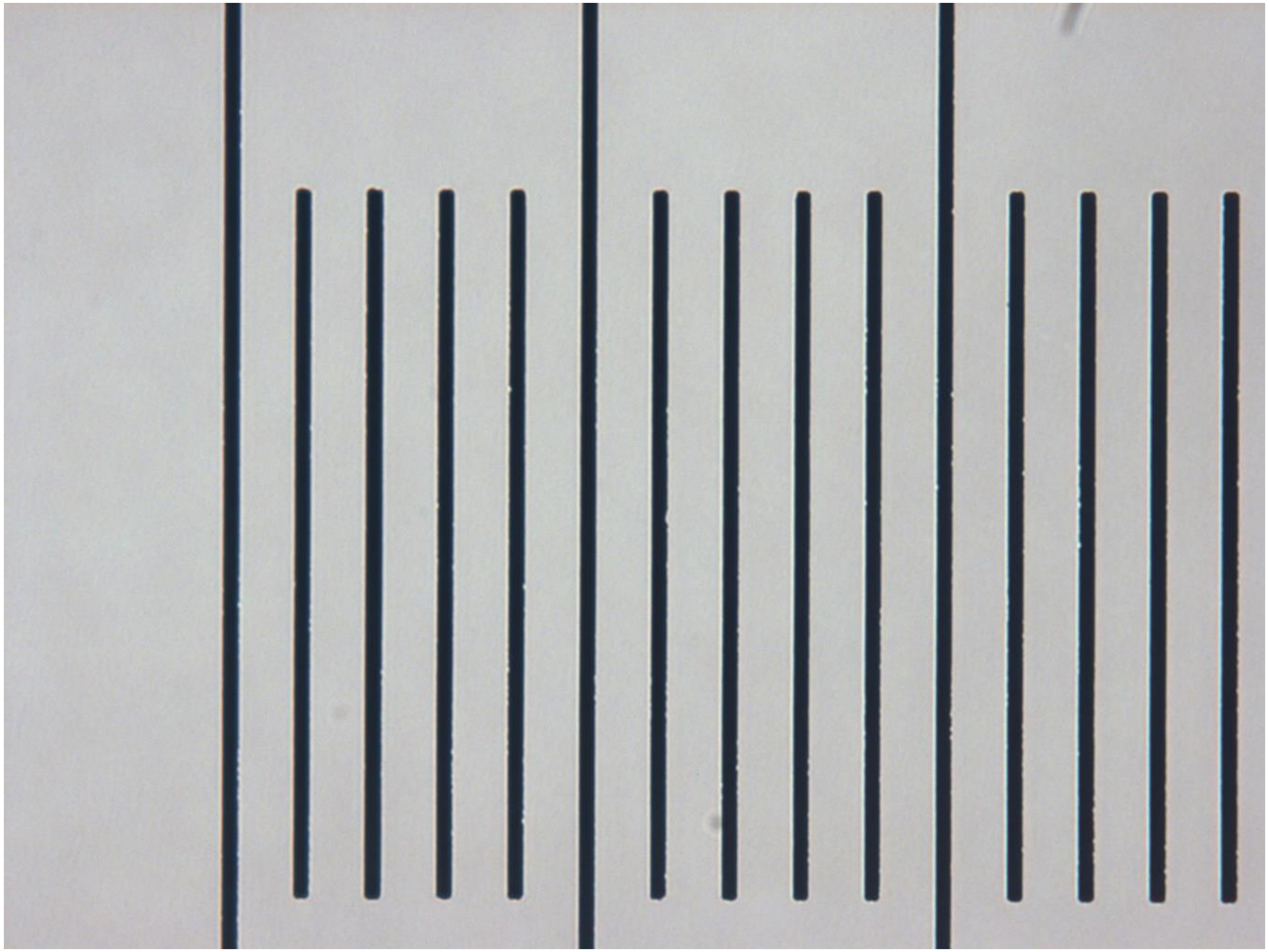

40x magnification

Blaberus craniifer cuticle samples

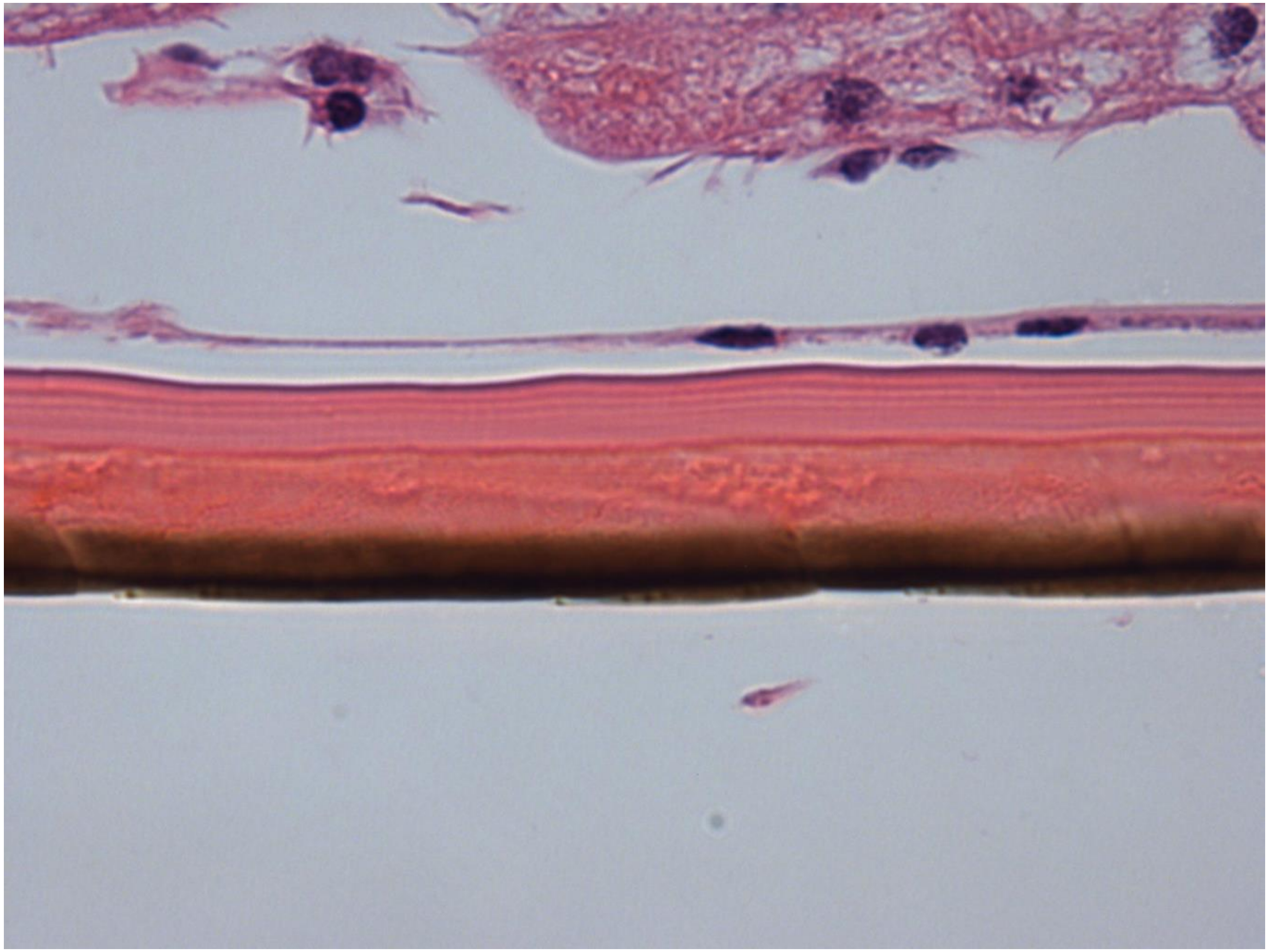

Blaberus craniifer **#1** **Abdominal cuticle** **40x magnification**

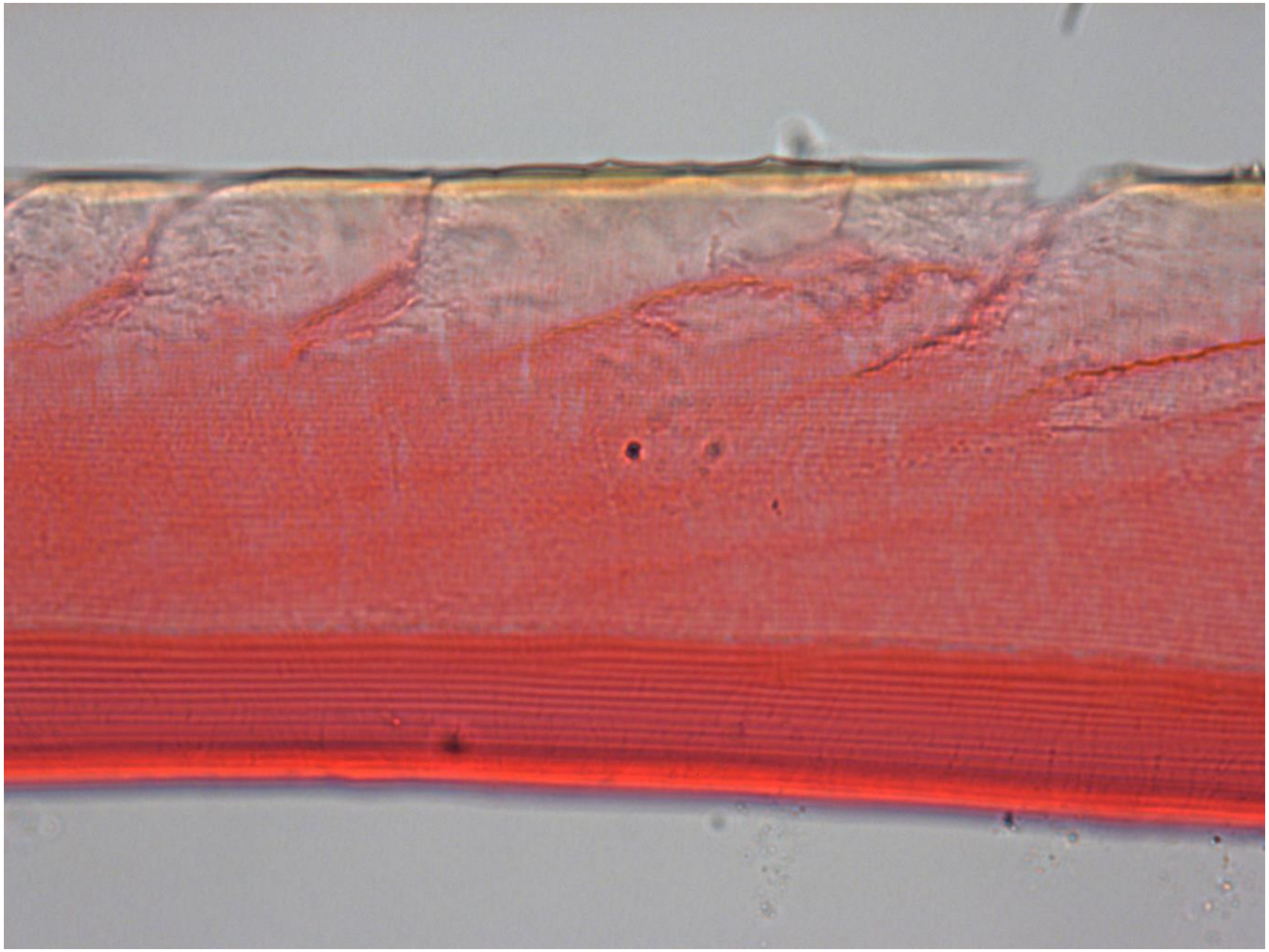

Blaberus craniifer #1 Thoracic cuticle 40x magnification

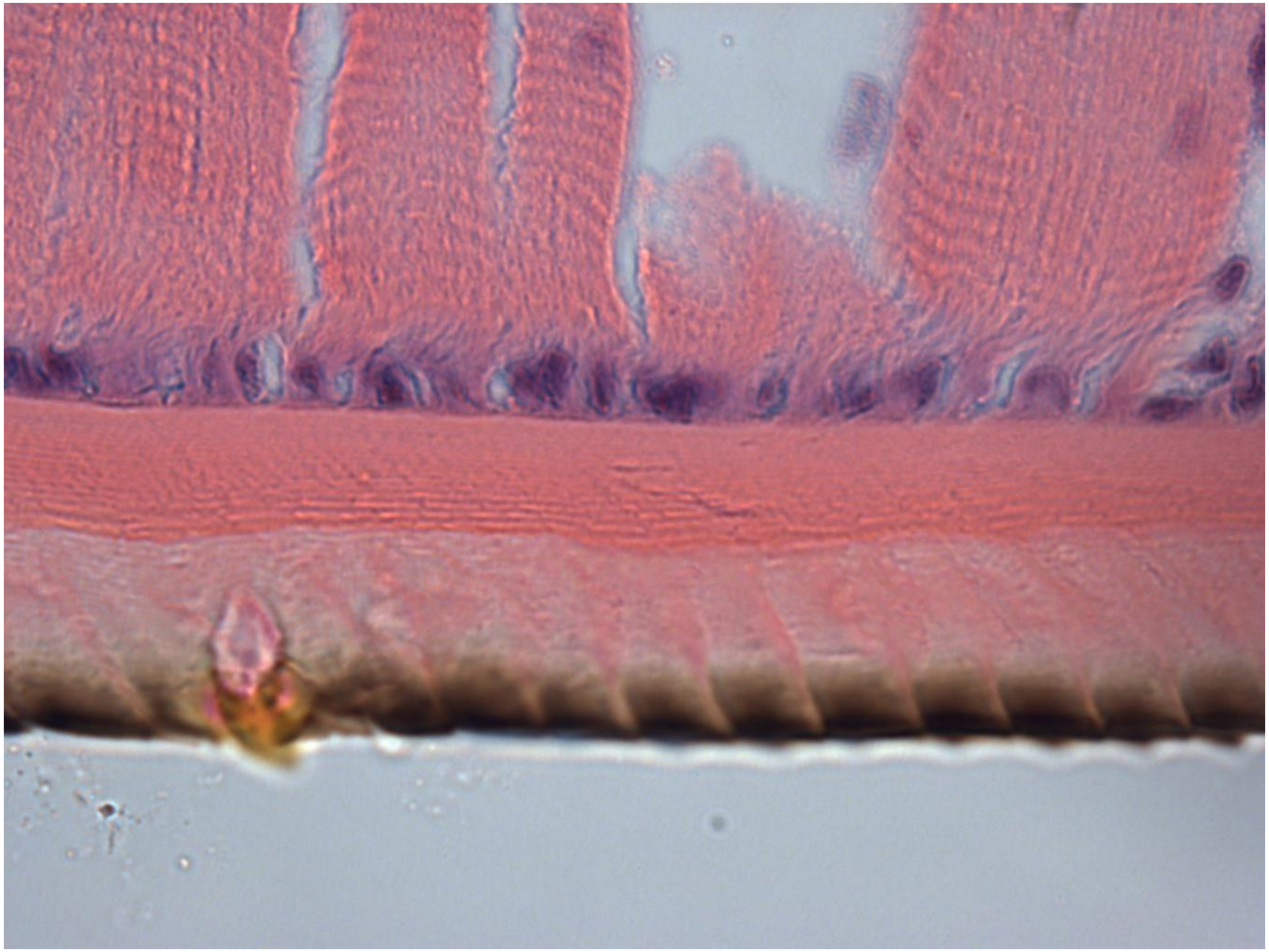

Blaberus craniifer **#2** **Abdominal cuticle** **40x magnification**

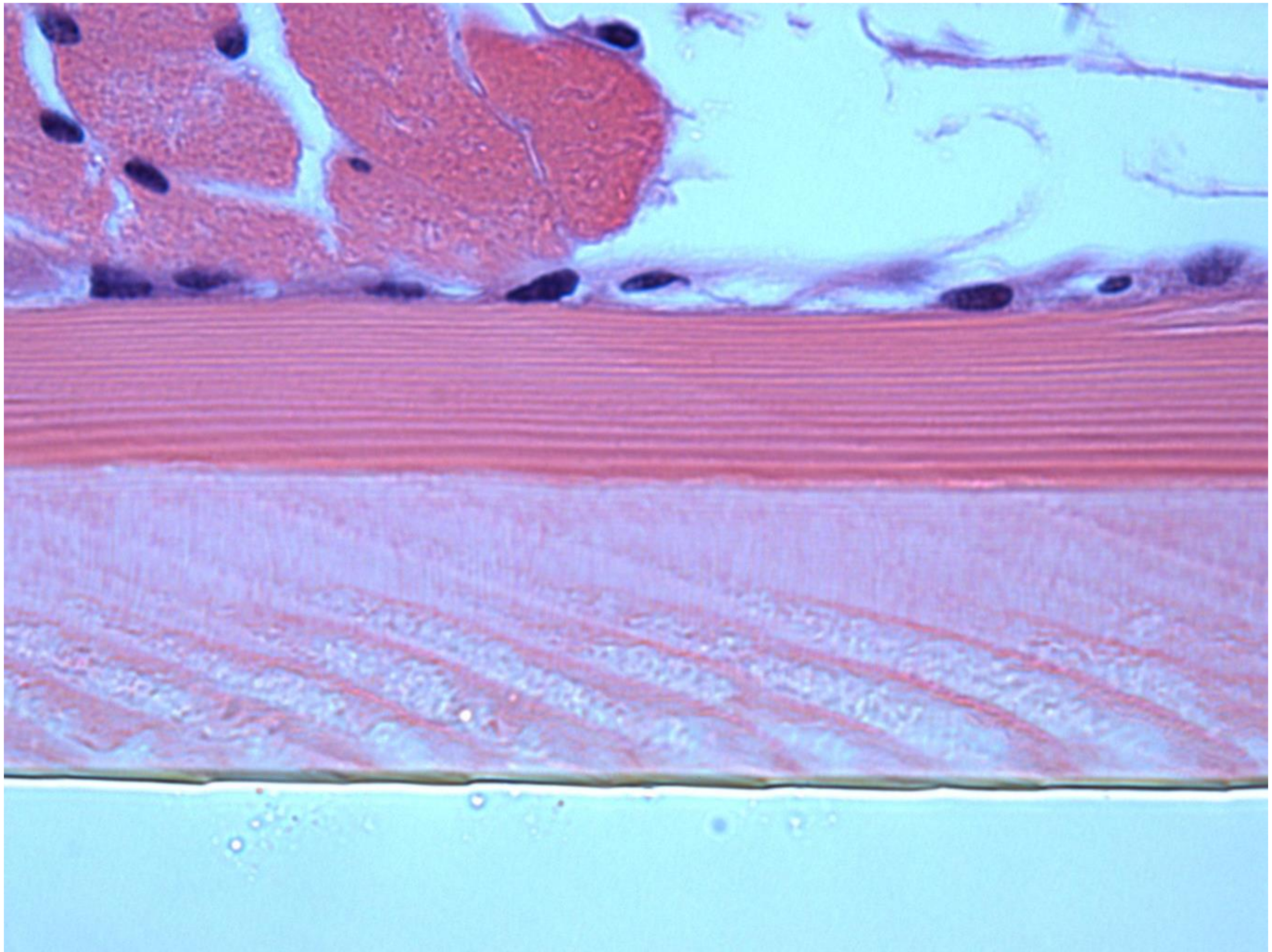

Blaberus craniifer #2 Thoracic cuticle 40x magnification

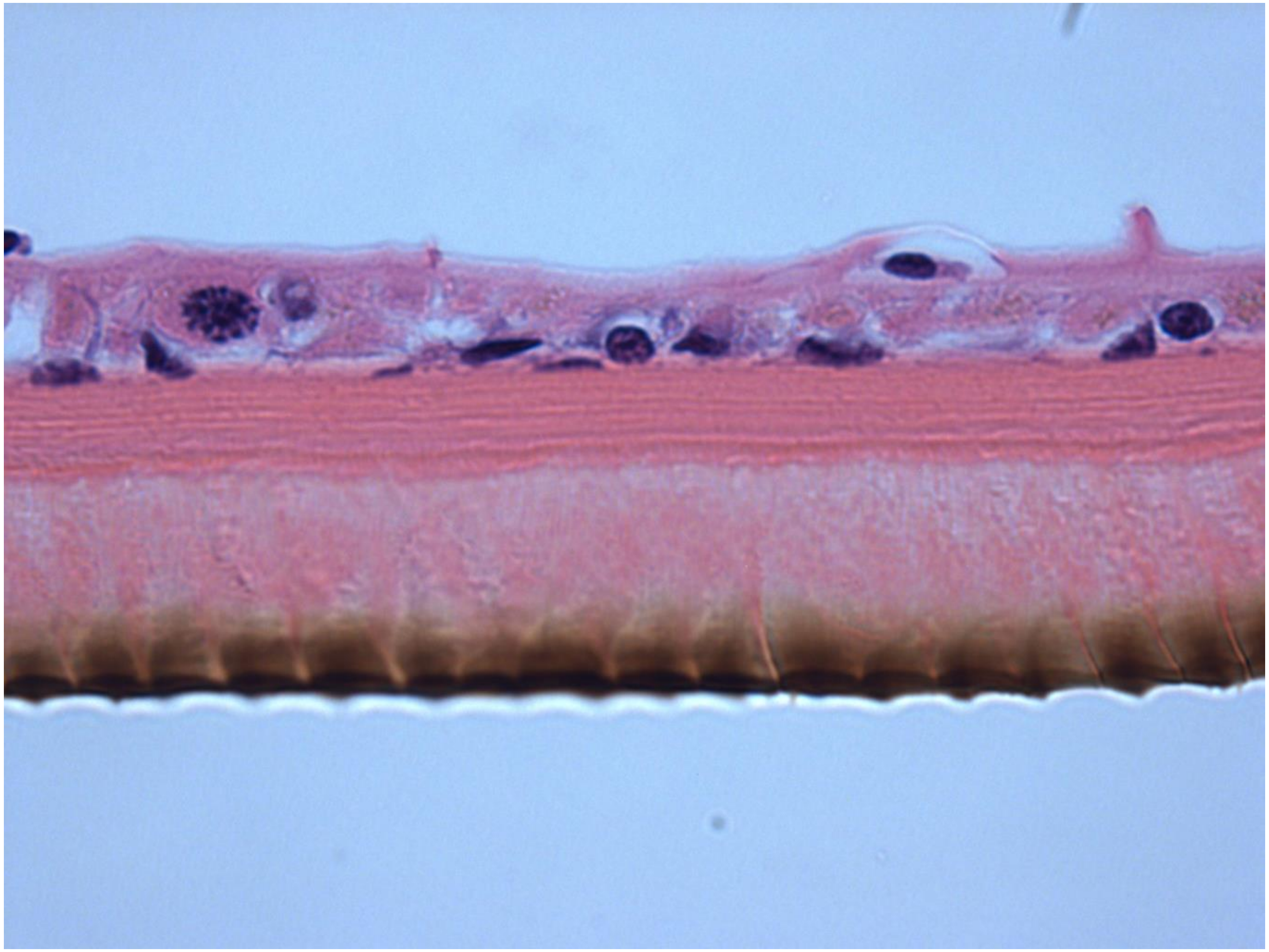

Blaberus craniifer **#3** **Abdominal cuticle** **40x magnification**

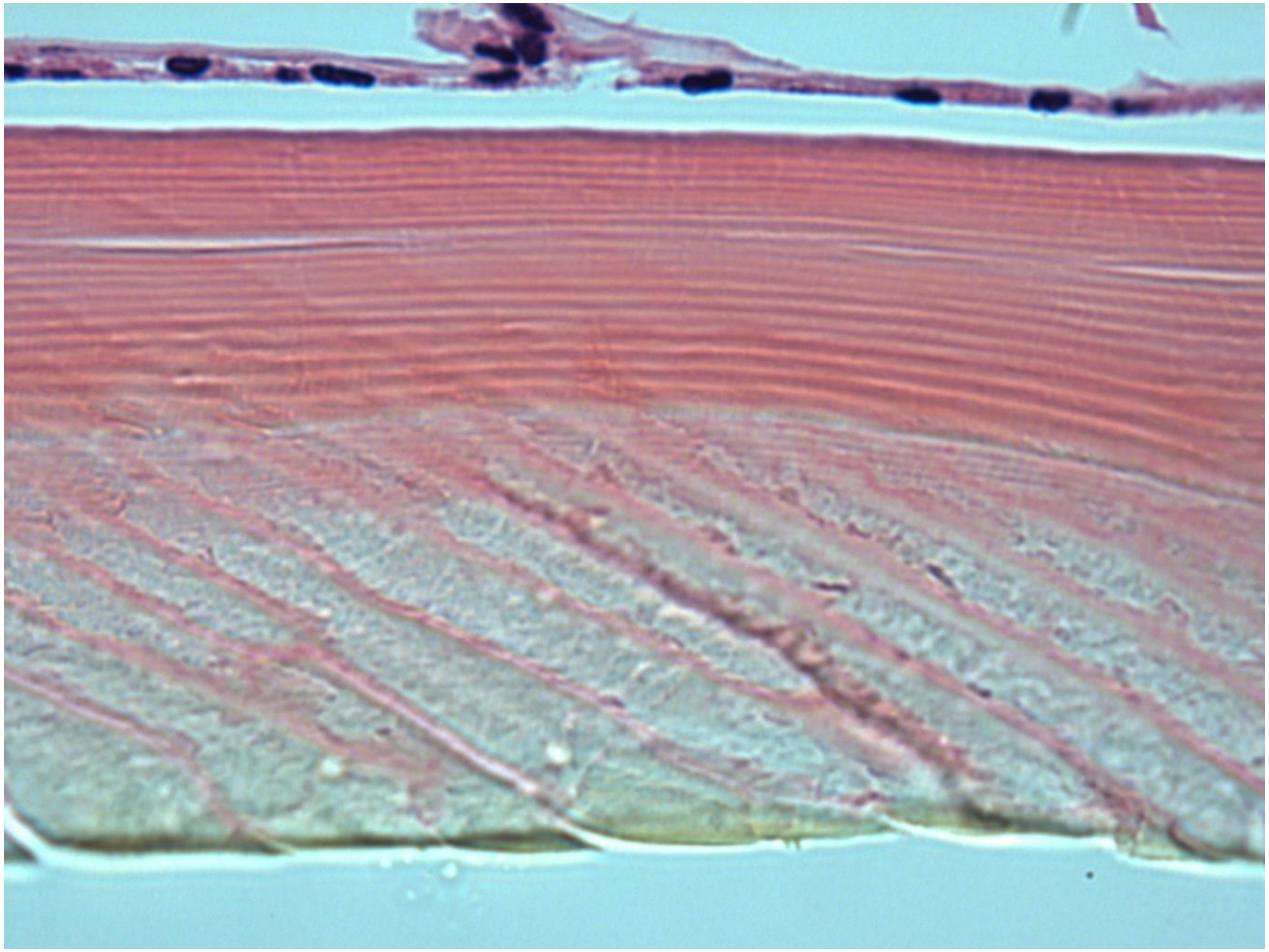

Blaberus craniifer #3 Thoracic cuticle 40x magnification

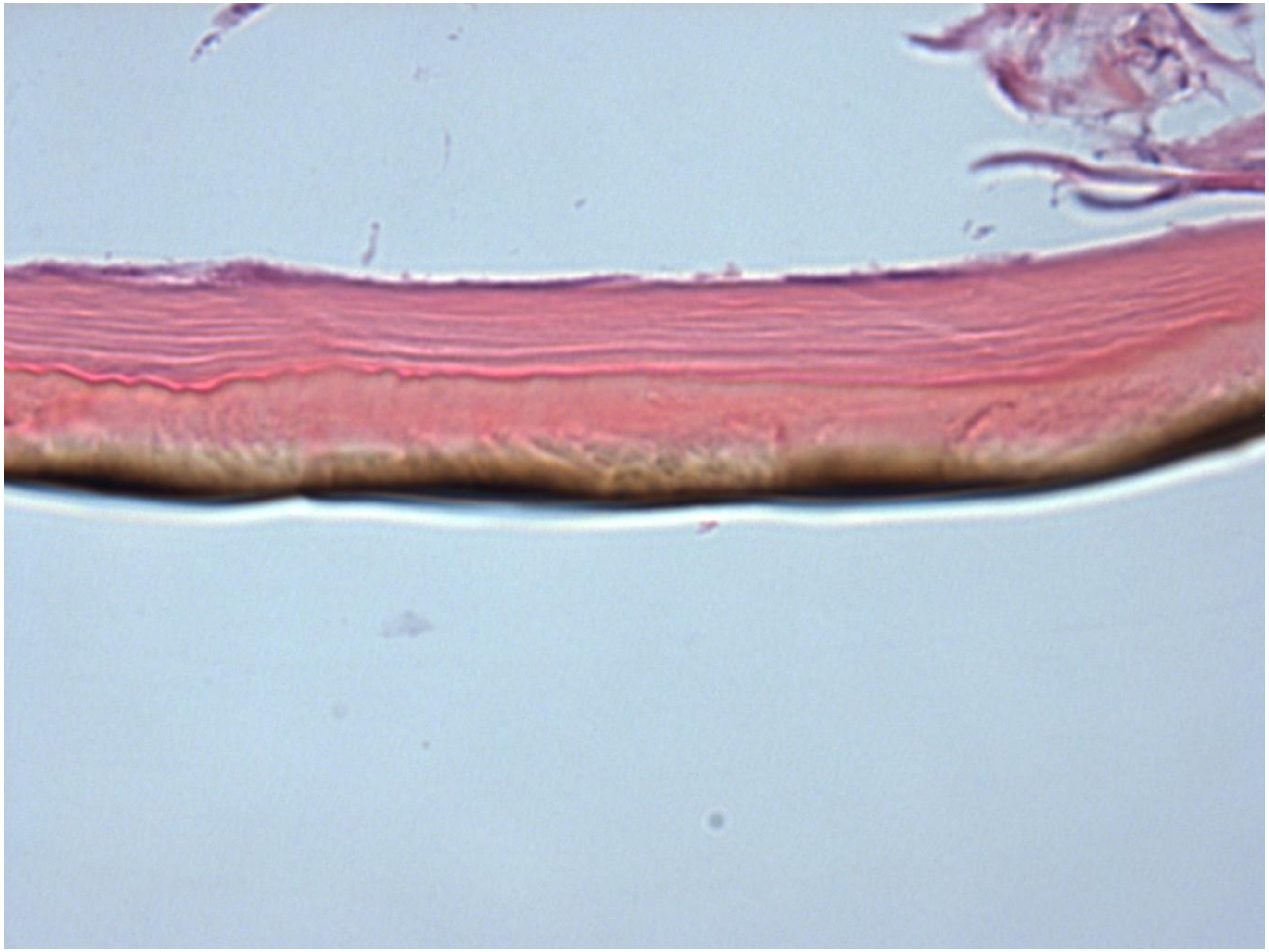

Blaberus craniifer #4 **Abdominal cuticle** **40x magnification**

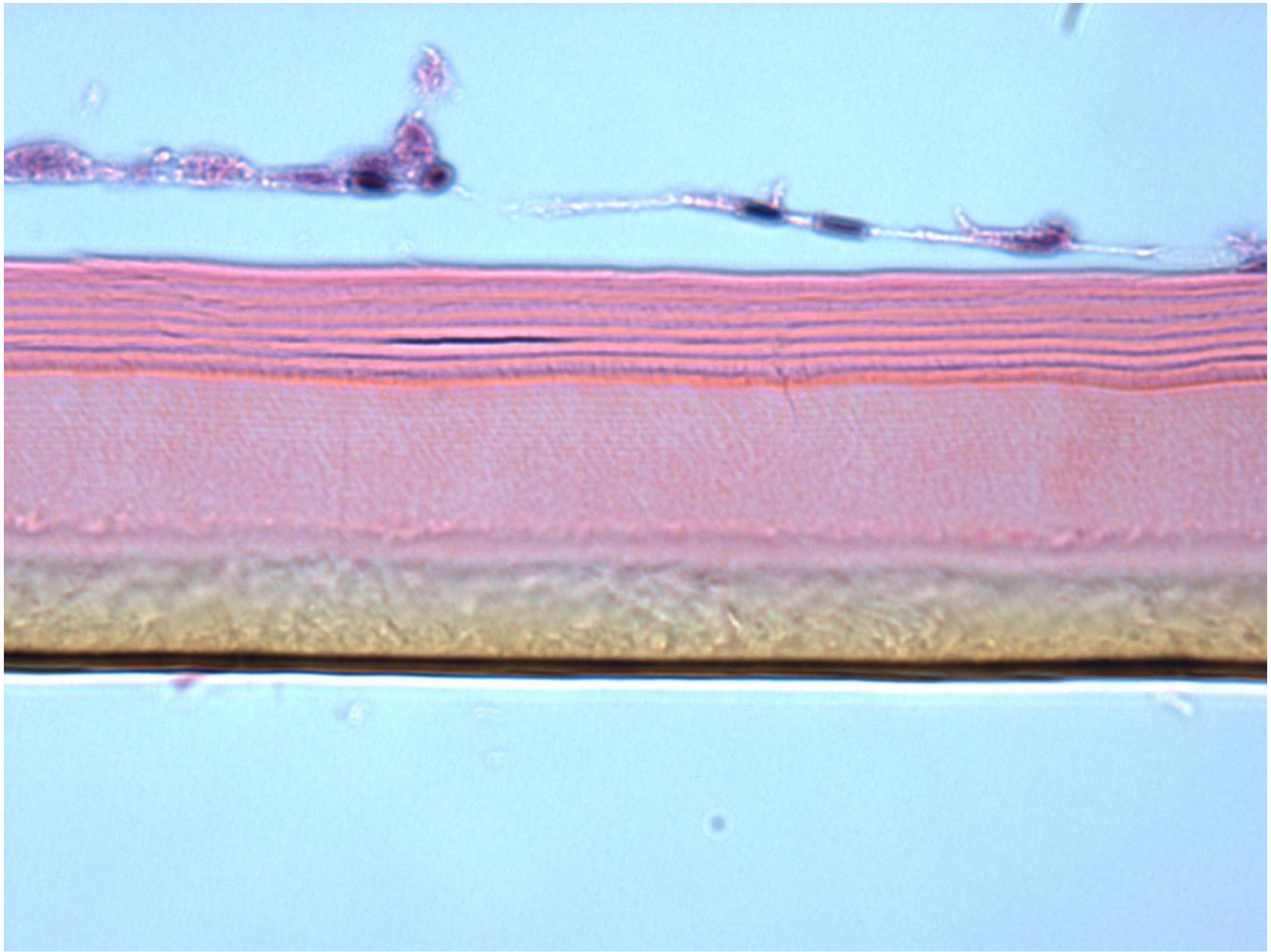

Blaberus craniifer #4　　Thoracic cuticle　　40x magnification

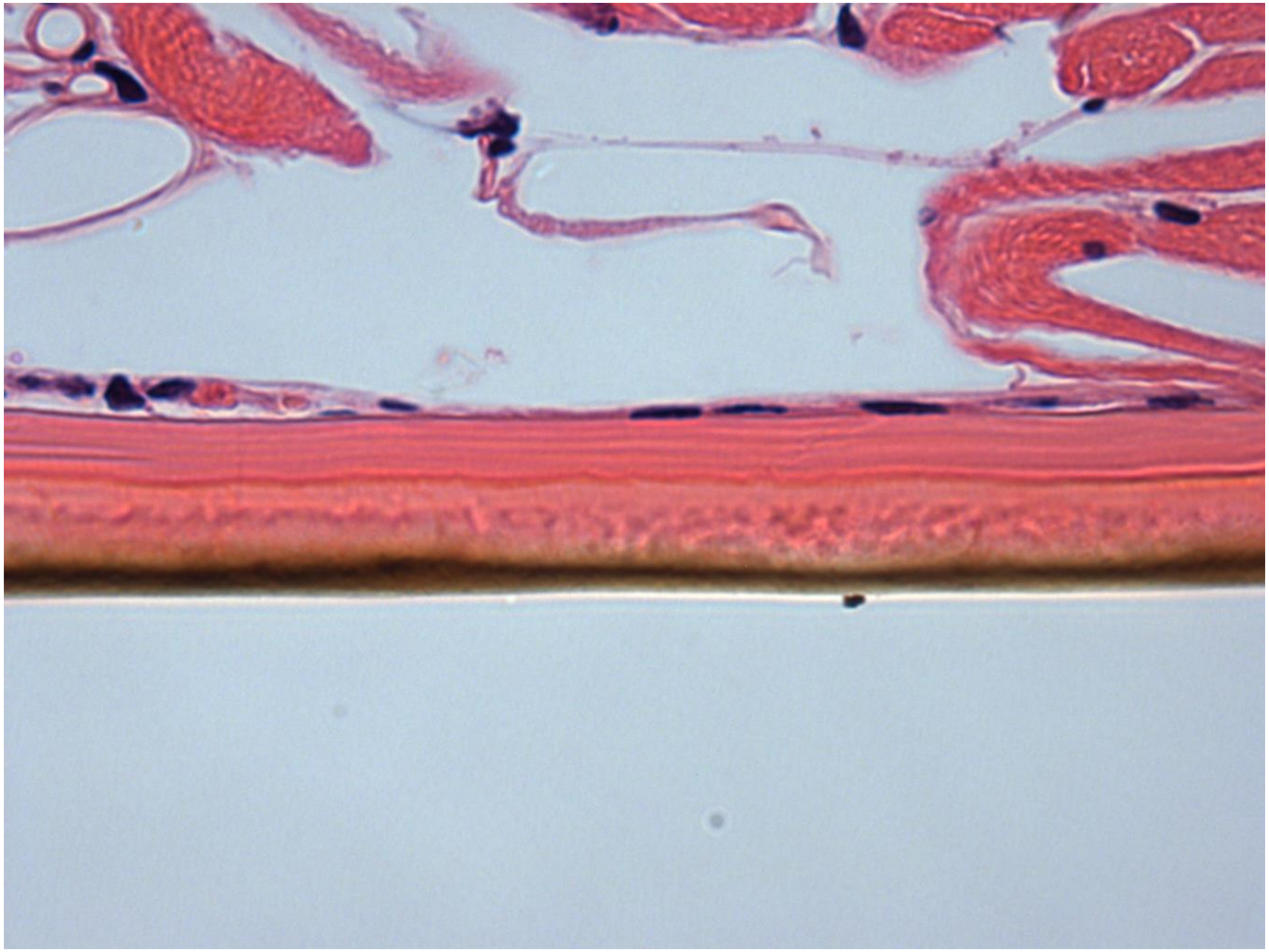

Blaberus craniifer **#5** **Abdominal cuticle** **40x magnification**

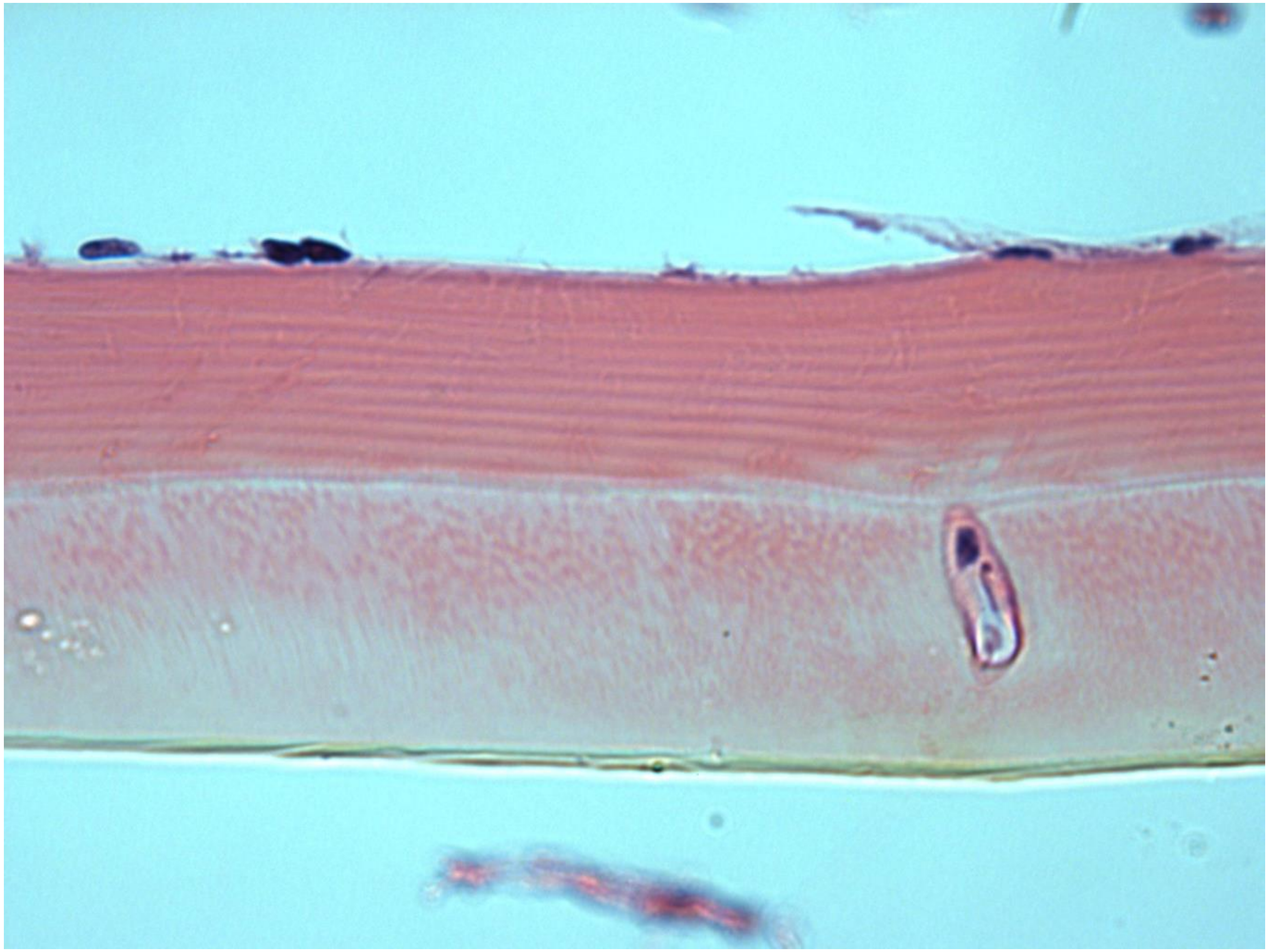

Blaberus craniifer #5 **Thoracic cuticle** **40x magnification**

	A	B	C	D	E	F	G	H	I
1	***Blaberus craniifer***								
2									
3		40x	**Thickness**	**ABDOMEN**	**BC#1_abd_40x**	**BC#2_abd_40x**	**BC#3_abd_40x**	**BC#4_abd_40x**	**BC#5_abd_40x**
4	Distance in pixels	1624	**(microns)**	(1) Cuticle	31.55	44.31	44.83	30.86	25.34
5	Known Distance	140 microns		(2) Procuticle	27.76	41.72	41.9	27.76	21.55
6		11.6 pixels/micron		(3) Endocuticle	11.21	16.38	14.48	13.62	10.52
7				Exocuticle	16.55	25.34	27.42	14.14	11.03
8				Epicuticle	3.79	2.59	2.93	3.1	3.79
9									
10		20x		**THORAX**	**BC#1_thor_40x**	**BC#2_thor_40x**	**BC#3_thor_40x**	**BC#4_thor_40x**	**BC#5_thor_40x**
11	Distance in pixels	1682		(1) Cuticle	85.34	66.21	100.69	57.41	70
12	Known Distance	290 microns		(2) Procuticle	83.97	65.69	99.14	54.66	68.79
13		5.8 pixels/micron		(3) Endocuticle	20.52	24.48	38.79	16.55	28.79
14				Exocuticle	63.45	41.21	60.35	38.11	40
15				Epicuticle	1.37	0.52	1.55	2.75	1.21
16		10x							
17	Distance in pixels	1798	**Percentages**	**ABDOMEN**	**BC#1_abd_40x**	**BC#2_abd_40x**	**BC#3_abd_40x**	**BC#4_abd_40x**	**BC#5_abd_40x**
18	Known Distance	620 microns		Epicuticle	12.0	5.8	6.5	10.0	15.0
19		2.9 pixels/micron		Exocuticle	52.5	57.2	61.2	45.8	43.5
20				Endocuticle	35.5	37.0	32.3	44.1	41.5
21					100.0	100.0	100.0	100.0	100.0
22				Procuticle	88.0	94.2	93.5	90.0	85.0
23									
24				**THORAX**	**BC#1_thor_40x**	**BC#2_thor_40x**	**BC#3_thor_40x**	**BC#4_thor_40x**	**BC#5_thor_40x**
25				Epicuticle	1.6	0.8	1.5	4.8	1.7
26				Exocuticle	74.3	62.2	59.9	66.4	57.1
27				Endocuticle	24.0	37.0	38.5	28.8	41.1
28					100.0	100.0	100.0	100.0	100.0
29				Procuticle	98.4	99.2	98.5	95.2	98.3

Gromphadorhina potentosa cuticle samples

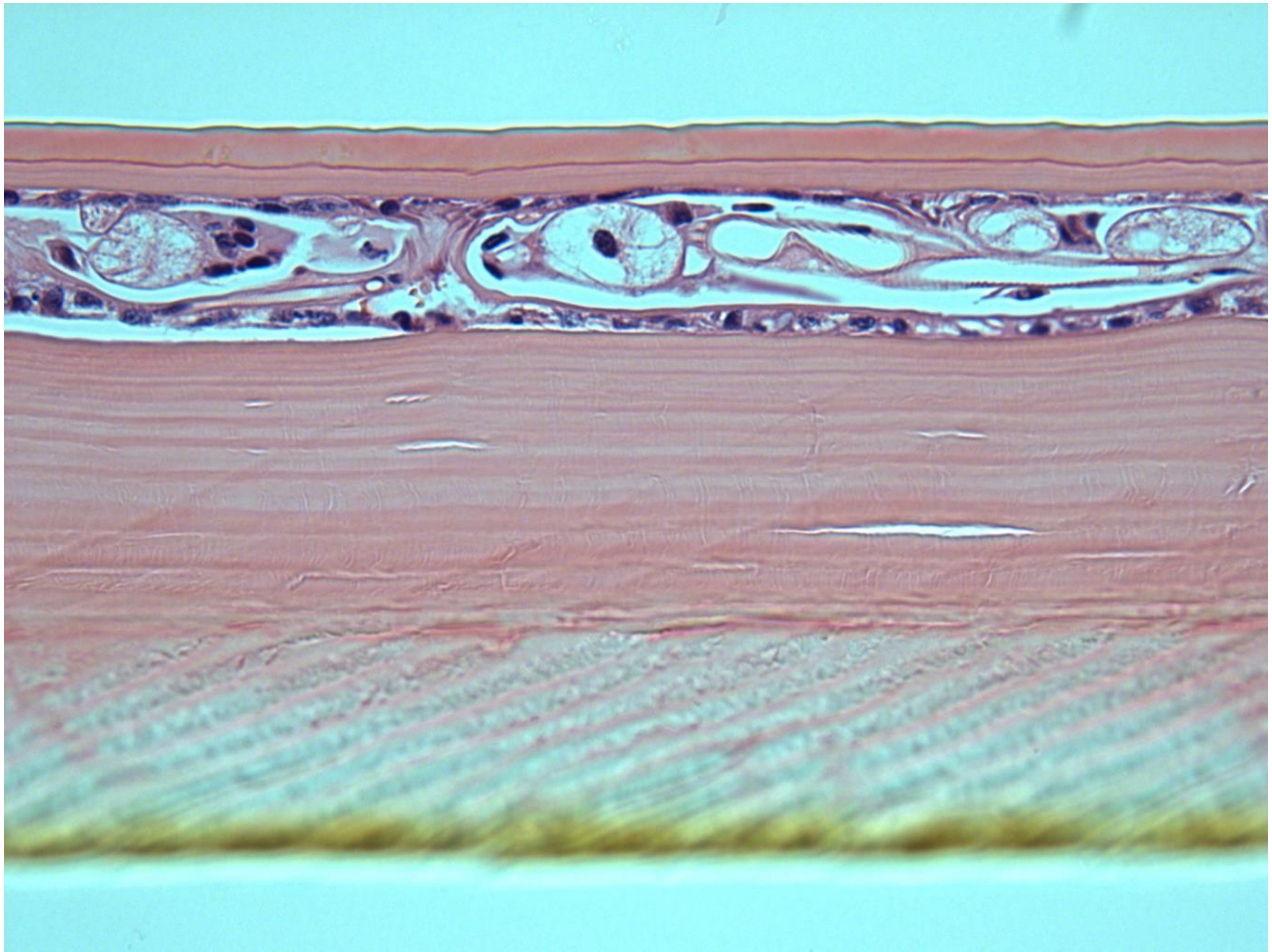

Gromphadorhina portentosa #1 Abdominal cuticle 20x magnification

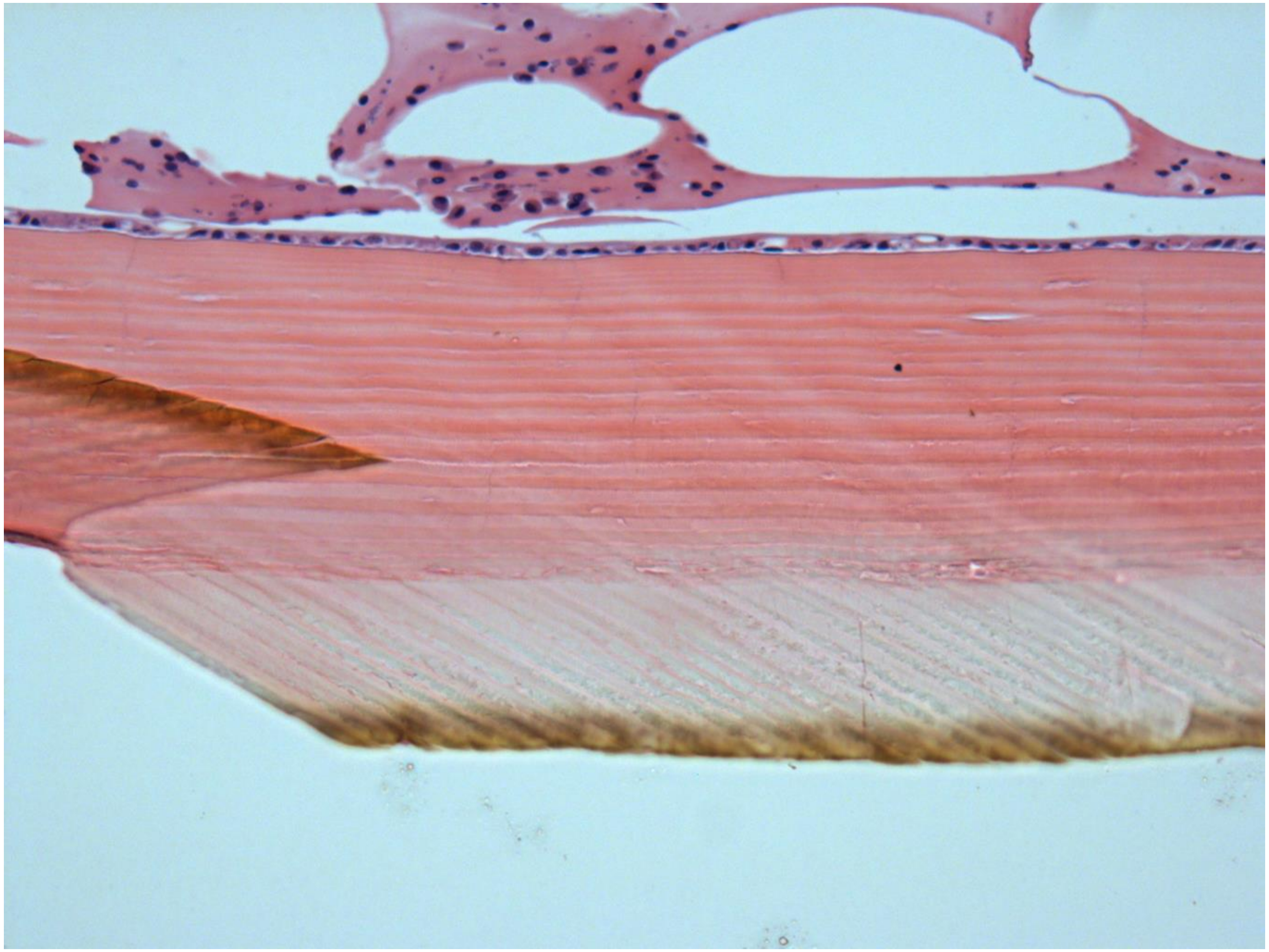

Gromphadorhina portentosa #1 Thoracic cuticle 10x magnification

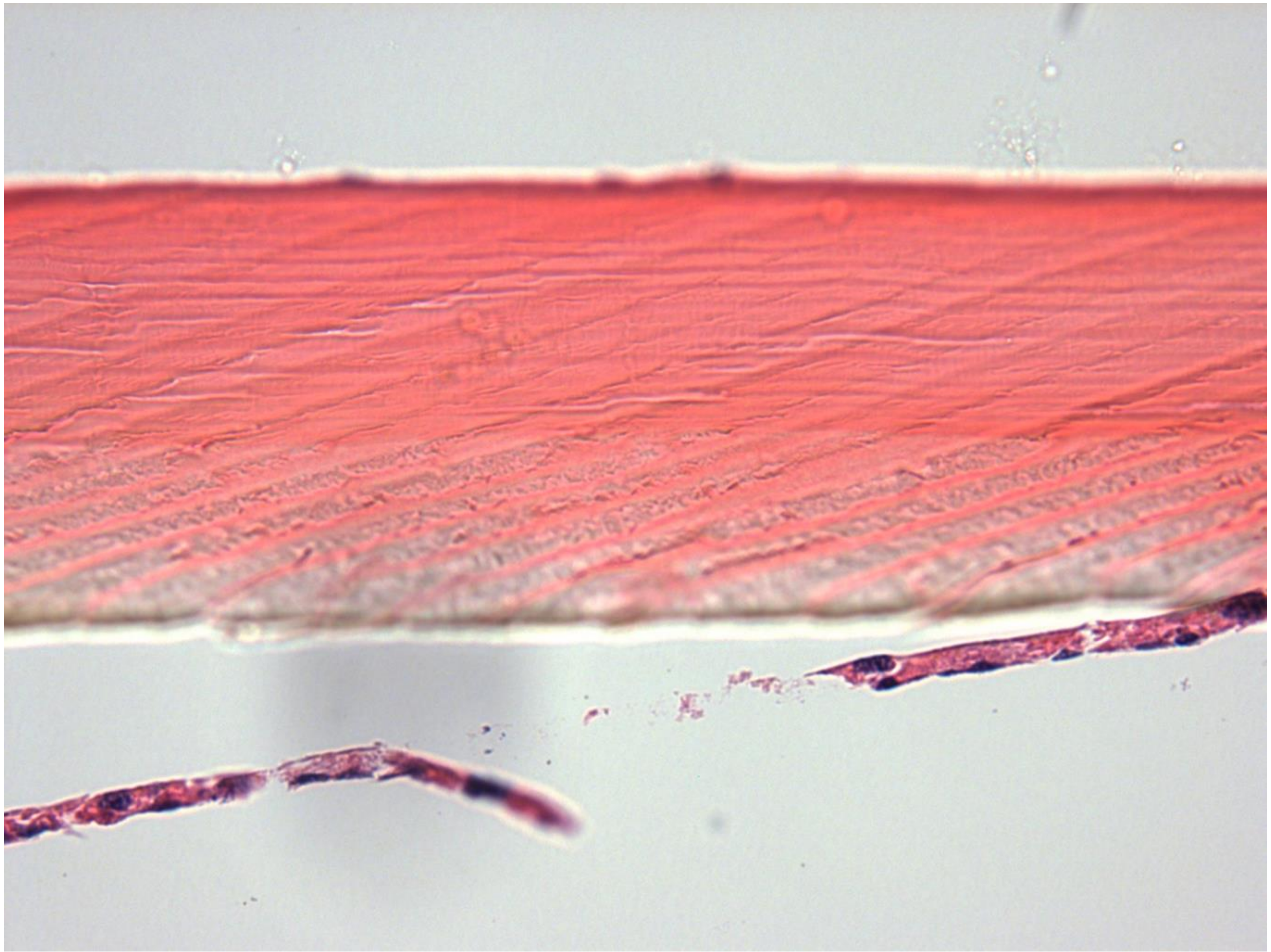

Gromphadorhina portentosa #2 Abdominal cuticle 20x magnification

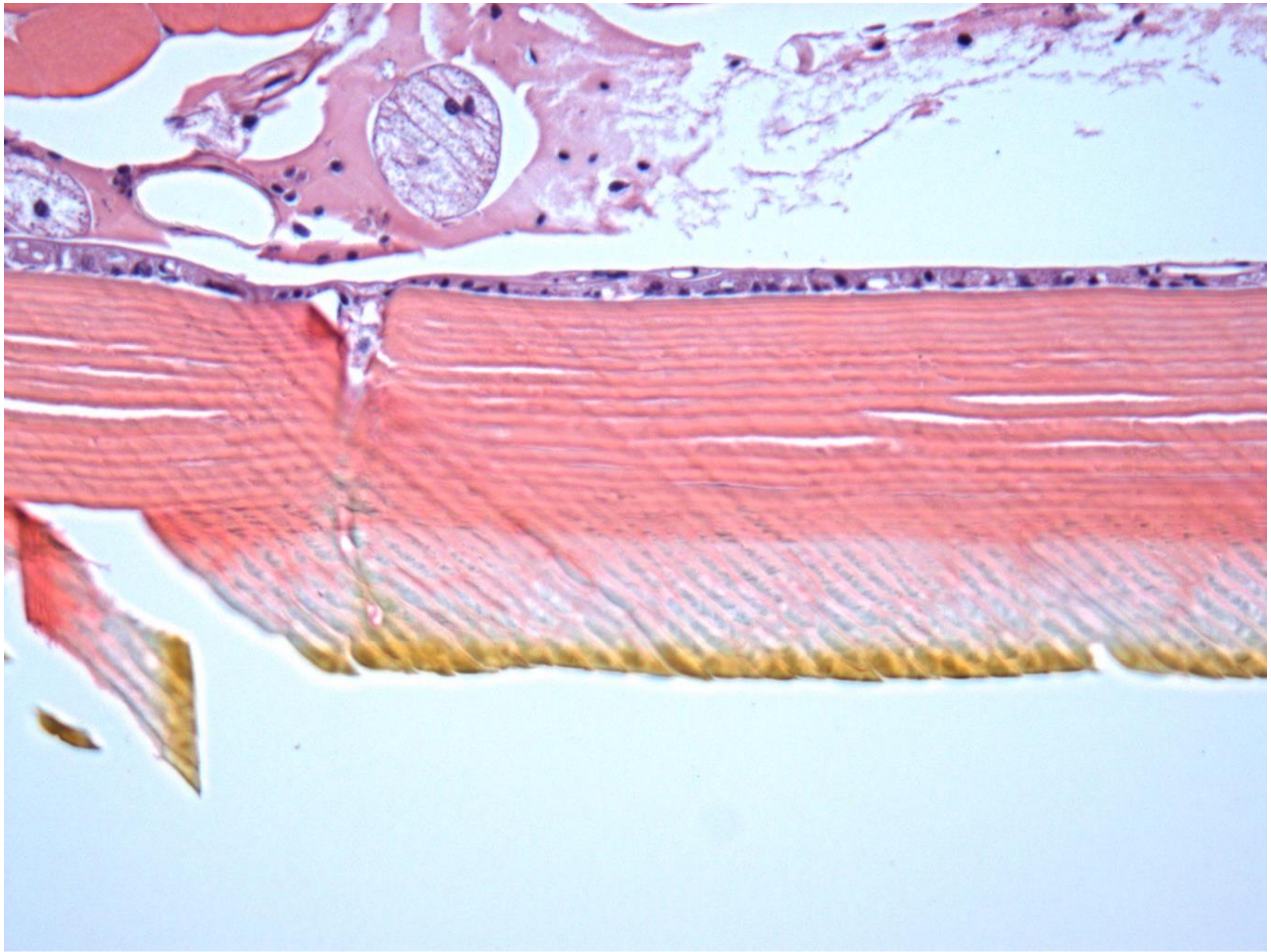

Gromphadorhina portentosa #2 **Thoracic cuticle** **10x magnification**

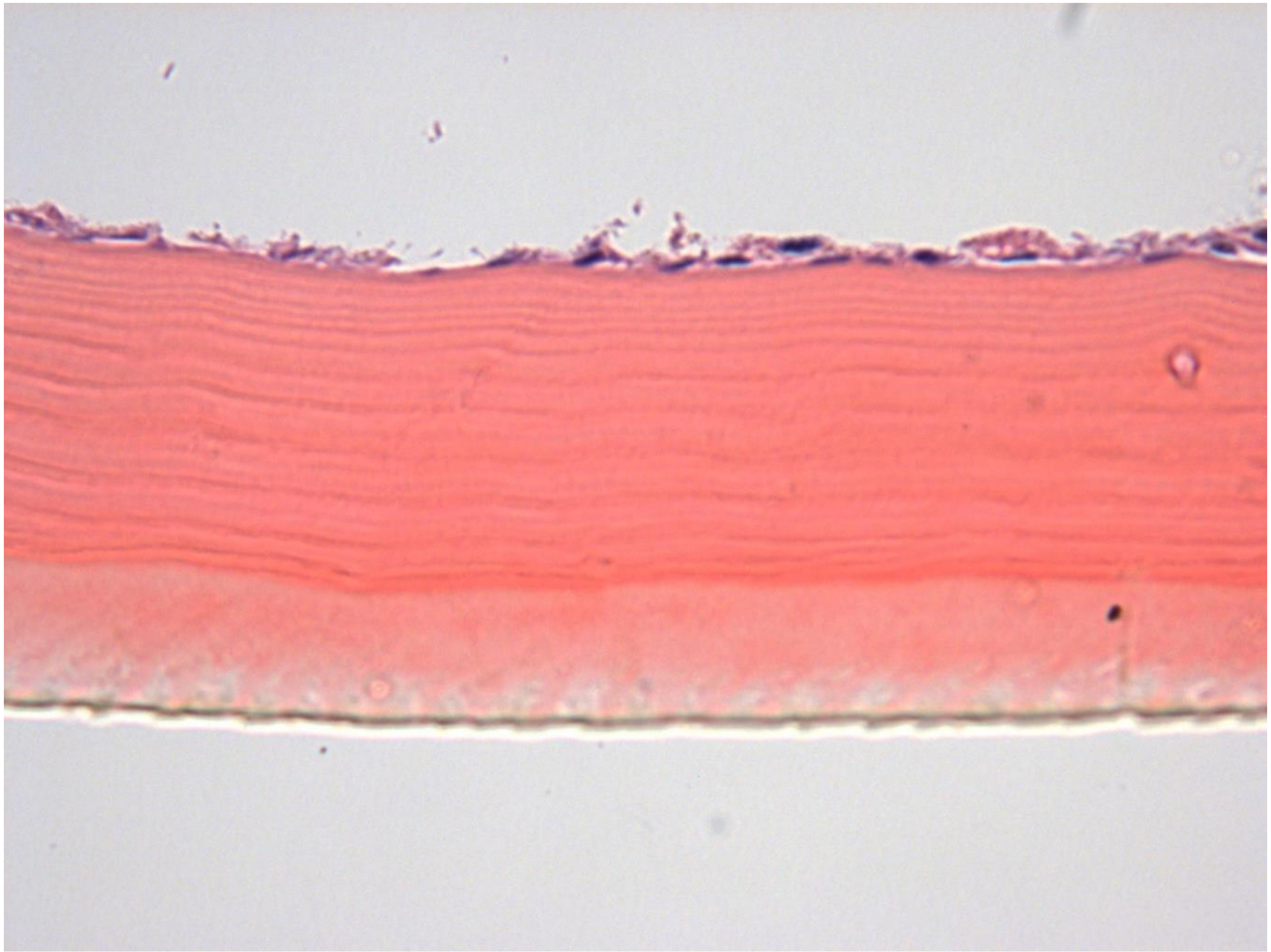

Gromphadorhina portentosa **#3** **Abdominal cuticle** **20x magnification**

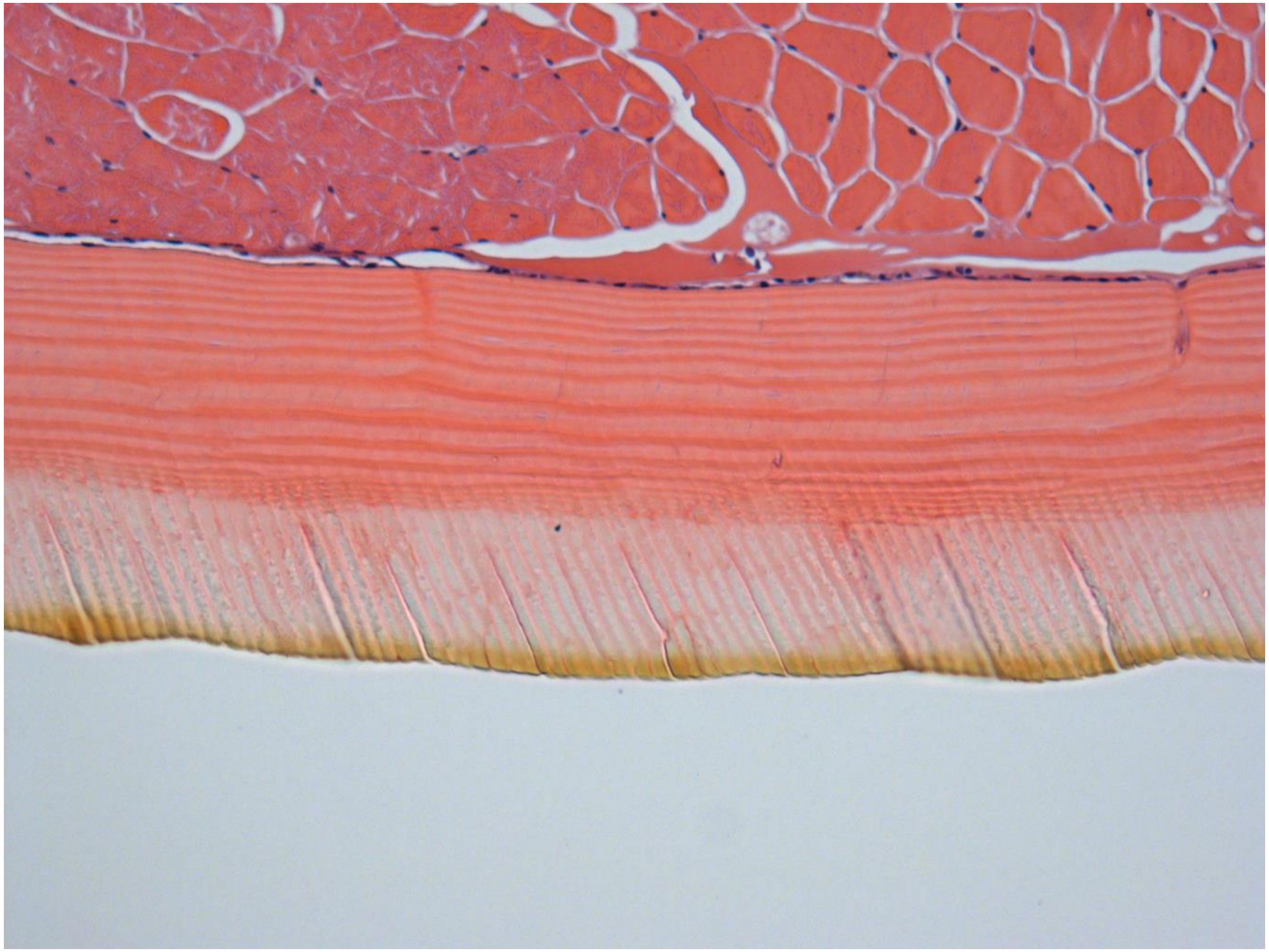

Gromphadorhina portentosa #3 **Thoracic cuticle** **10x magnification**

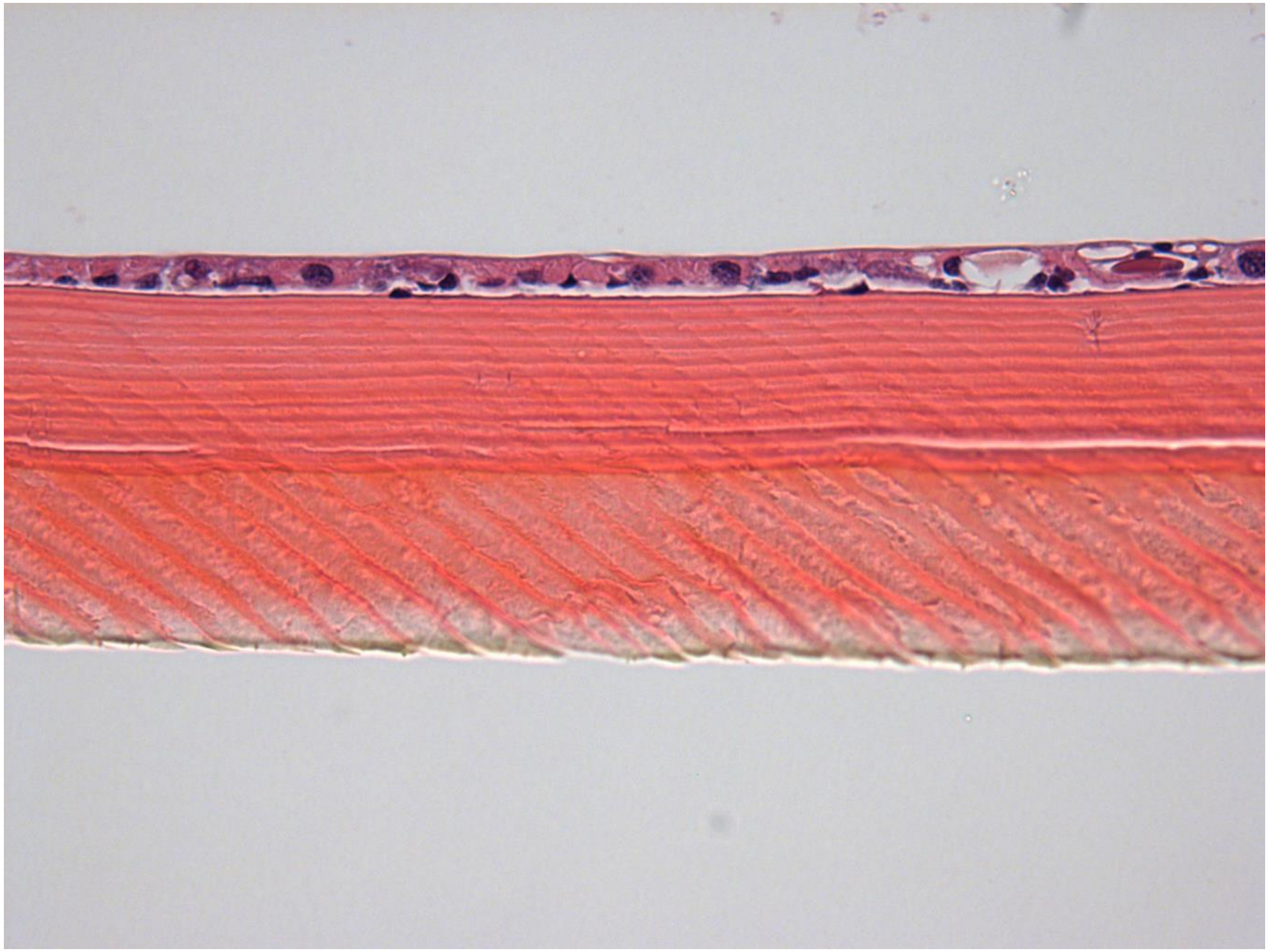

Gromphadorhina portentosa #4 **Abdominal cuticle** **20x magnification**

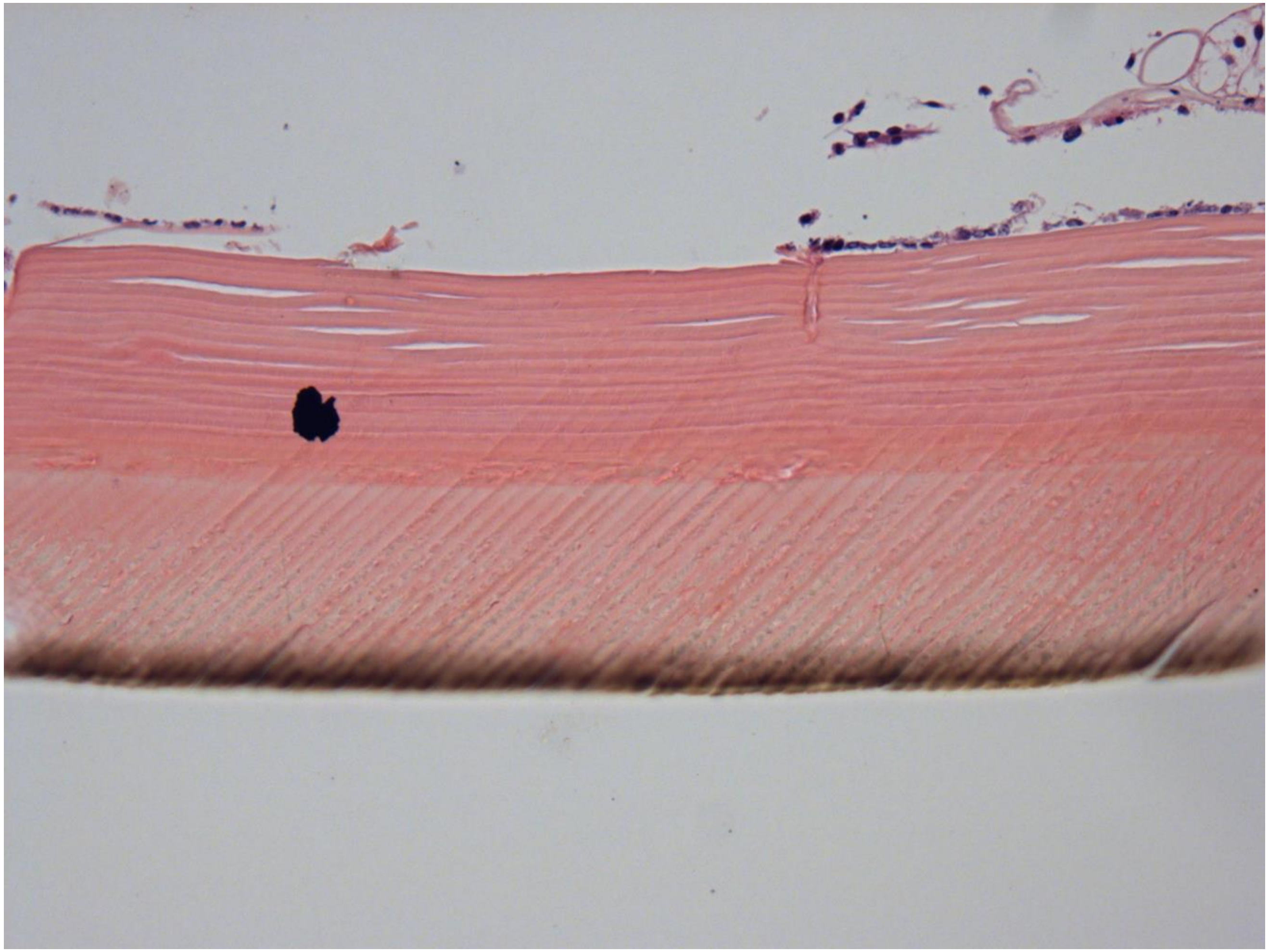

Gromphadorhina portentosa #4 Thoracic cuticle 10x magnification

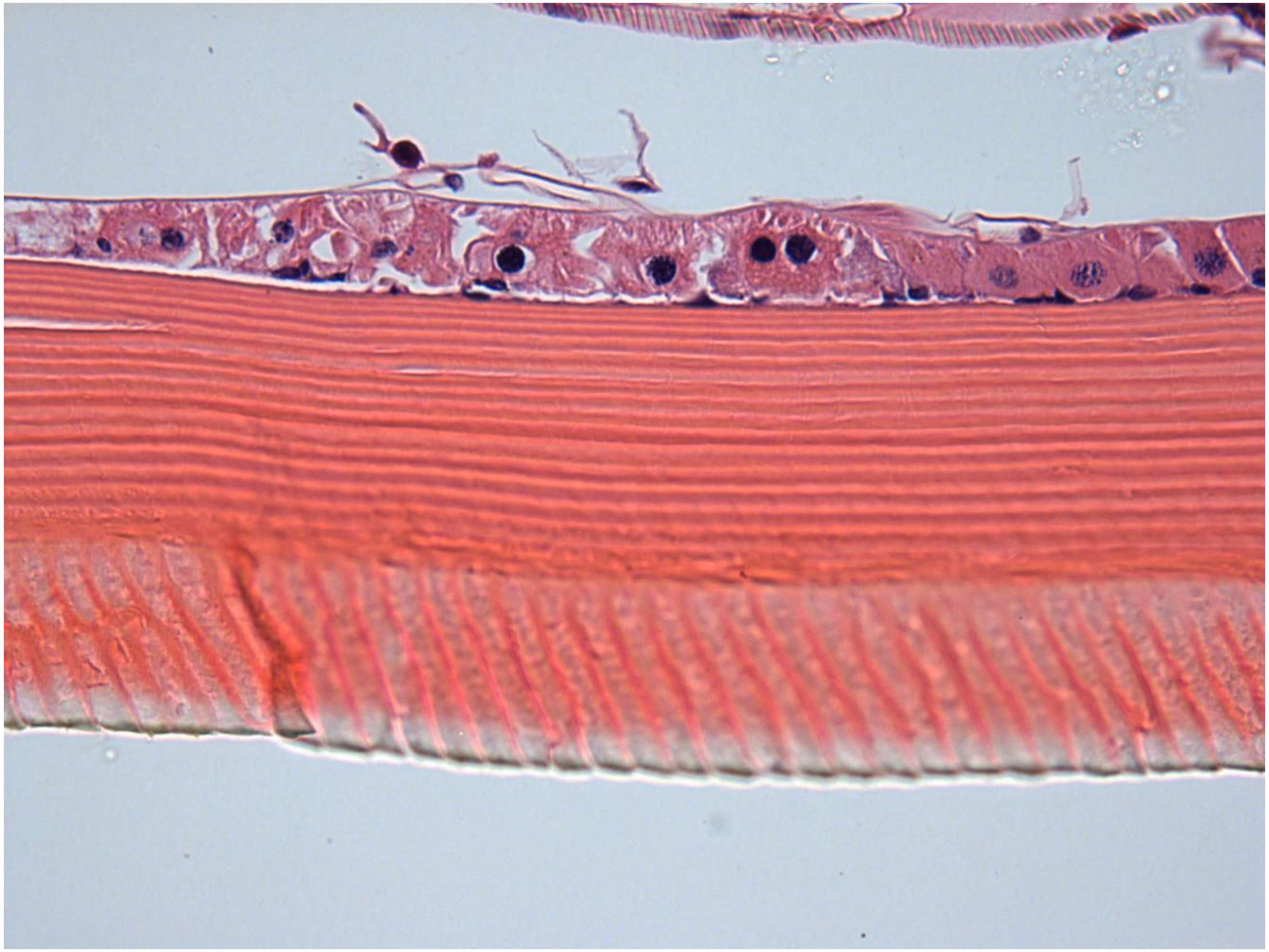

Gromphadorhina portentosa #5 **Abdominal cuticle** **20x magnification**

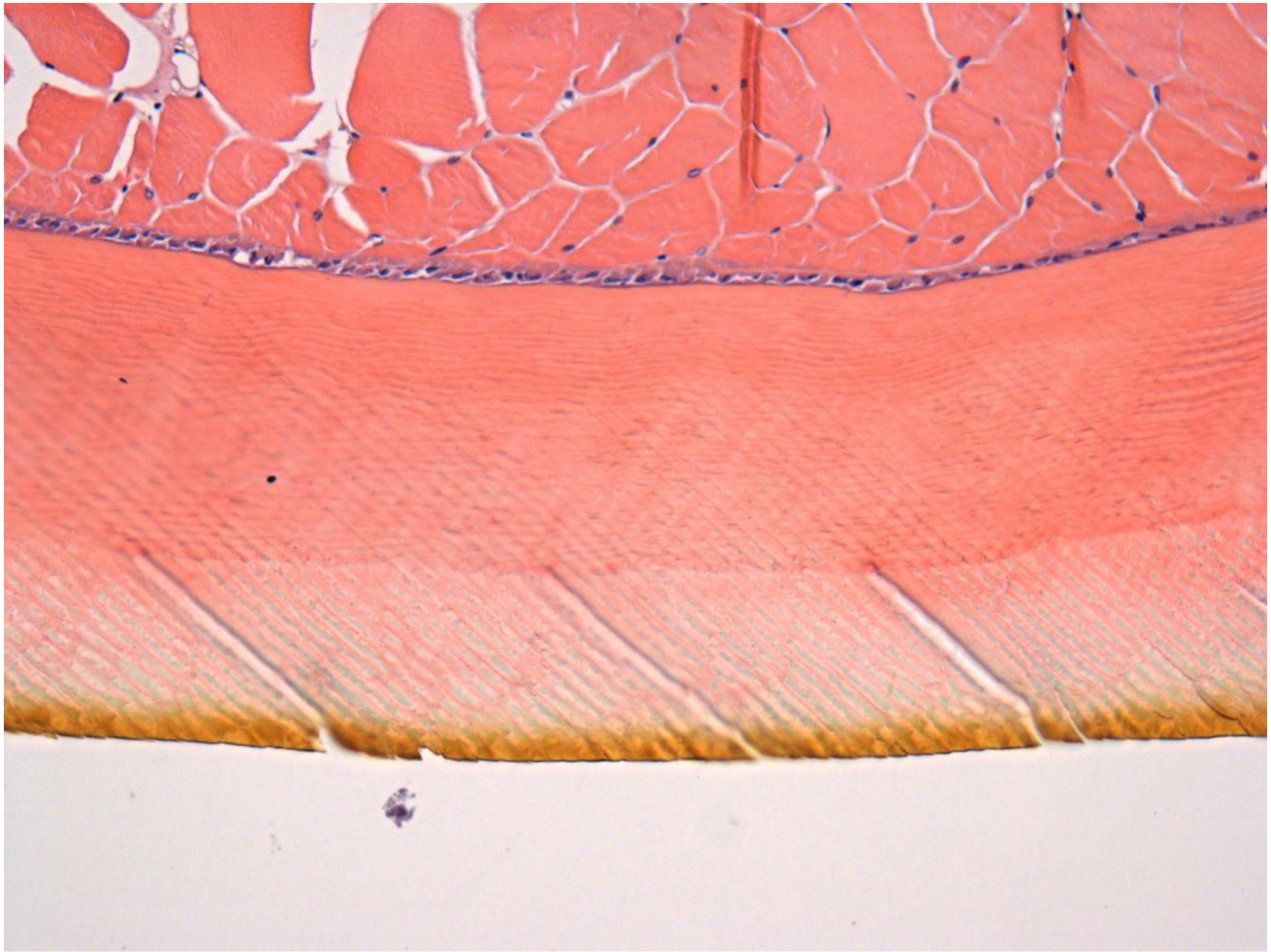

Gromphadorhina portentosa #5 Thoracic cuticle 10x magnification

	A	B	C	D	E	F	G	H	I
1	***Gromphadorhina portentosa***								
2									
3		40x	**Thickness**	**ABDOMEN**	**GP#1_abd_20x**	**GP#2_abd_20x**	**GP#3_abd_20x**	**GP#4_abd_20x**	**GP#5_abd_20x**
4	Distance in pixels	1624	**(microns)**	(1) Cuticle	147.24	123.1	128.28	100.34	132.07
5	Known Distance	140 microns		(2) Procuticle	141.72	119.66	124.48	97.59	128.62
6		11.6 pixels/micron		(3) Endocuticle	83.1	70	87.59	50.69	77.24
7				Exocuticle	58.62	49.66	36.89	46.9	51.38
8				Epicuticle	5.52	3.44	3.8	2.75	3.45
9									
10		20x		**THORAX**	**GP#1_thor_10x**	**GP#2_thor_10x**	**GP#3_thor_10x**	**GP#4_thor_10x**	**GP#5_thor_10x**
11	Distance in pixels	1682		(1) Cuticle	284.14	212.41	280	233.1	267.59
12	Known Distance	290 microns		(2) Procuticle	281.38	208.97	277.24	223.45	264.83
13		5.8 pixels/micron		(3) Endocuticle	180.69	135.17	173.1	120	161.38
14				Exocuticle	100.69	73.8	104.14	103.45	103.45
15				Epicuticle	2.76	3.44	2.76	9.65	2.76
16		10x							
17	Distance in pixels	1798	**Percentages**	**ABDOMEN**	**GP#1_abd_20x**	**GP#2_abd_20x**	**GP#3_abd_20x**	**GP#4_abd_20x**	**GP#5_abd_20x**
18	Known Distance	620 microns		Epicuticle	3.7	2.8	3.0	2.7	2.6
19		2.9 pixels/micron		Exocuticle	39.8	40.3	28.8	46.7	38.9
20				Endocuticle	56.4	56.9	68.3	50.5	58.5
21					100.0	100.0	100.0	100.0	100.0
22				Procuticle	96.3	97.2	97.0	97.3	97.4
23									
24				**THORAX**	**GP#1_thor_10x**	**GP#2_thor_10x**	**GP#3_thor_10x**	**GP#4_thor_10x**	**GP#5_thor_10x**
25				Epicuticle	1.0	1.6	1.0	4.1	1.0
26				Exocuticle	35.4	34.7	37.2	44.4	38.7
27				Endocuticle	63.6	63.6	61.8	51.5	60.3
28					100.0	100.0	100.0	100.0	100.0
29				Procuticle	99.0	98.4	99.0	95.9	99.0

Periplaneta americana cuticle samples

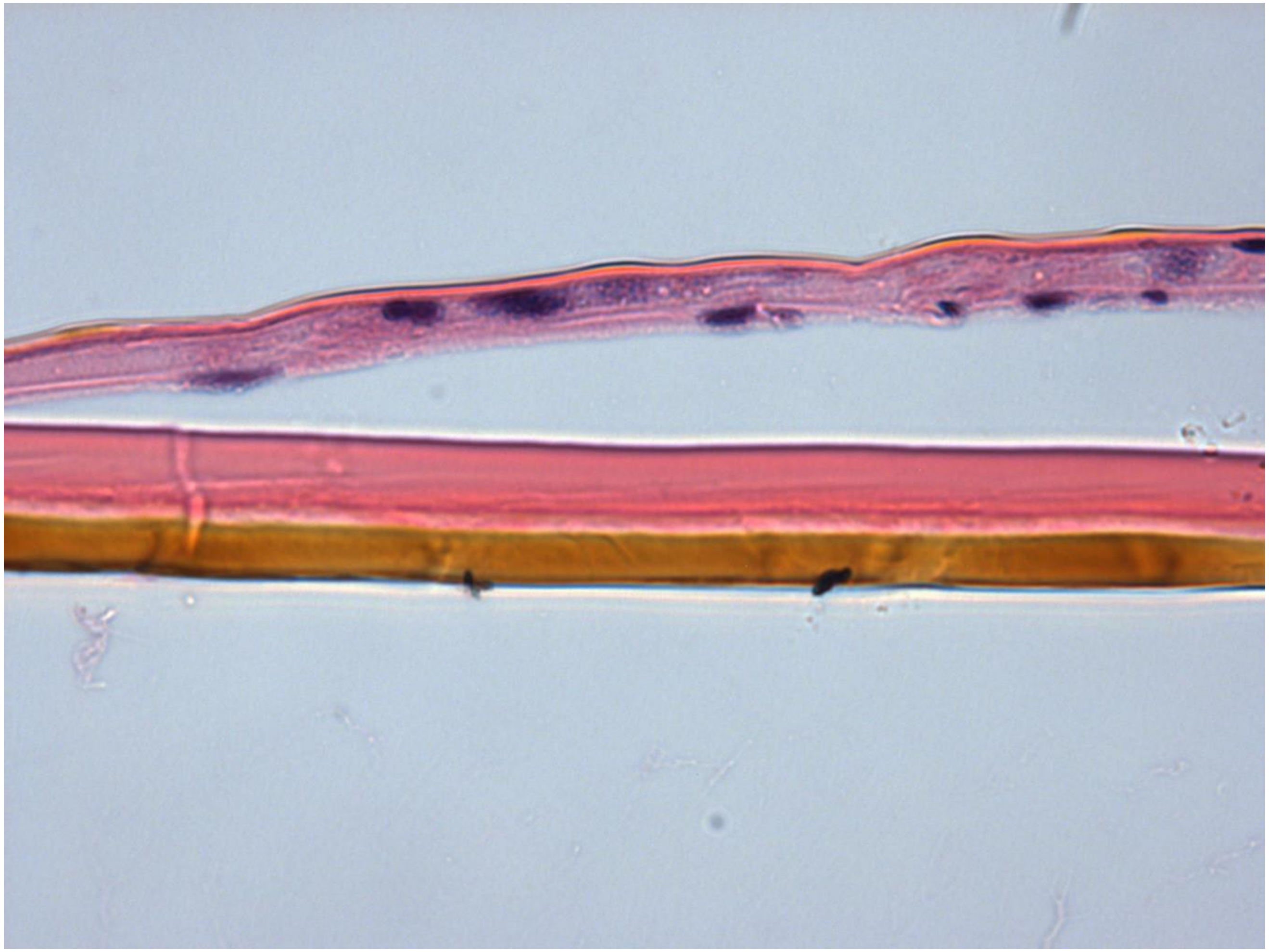

Periplaneta americana #1 Abdominal cuticle 40x magnification

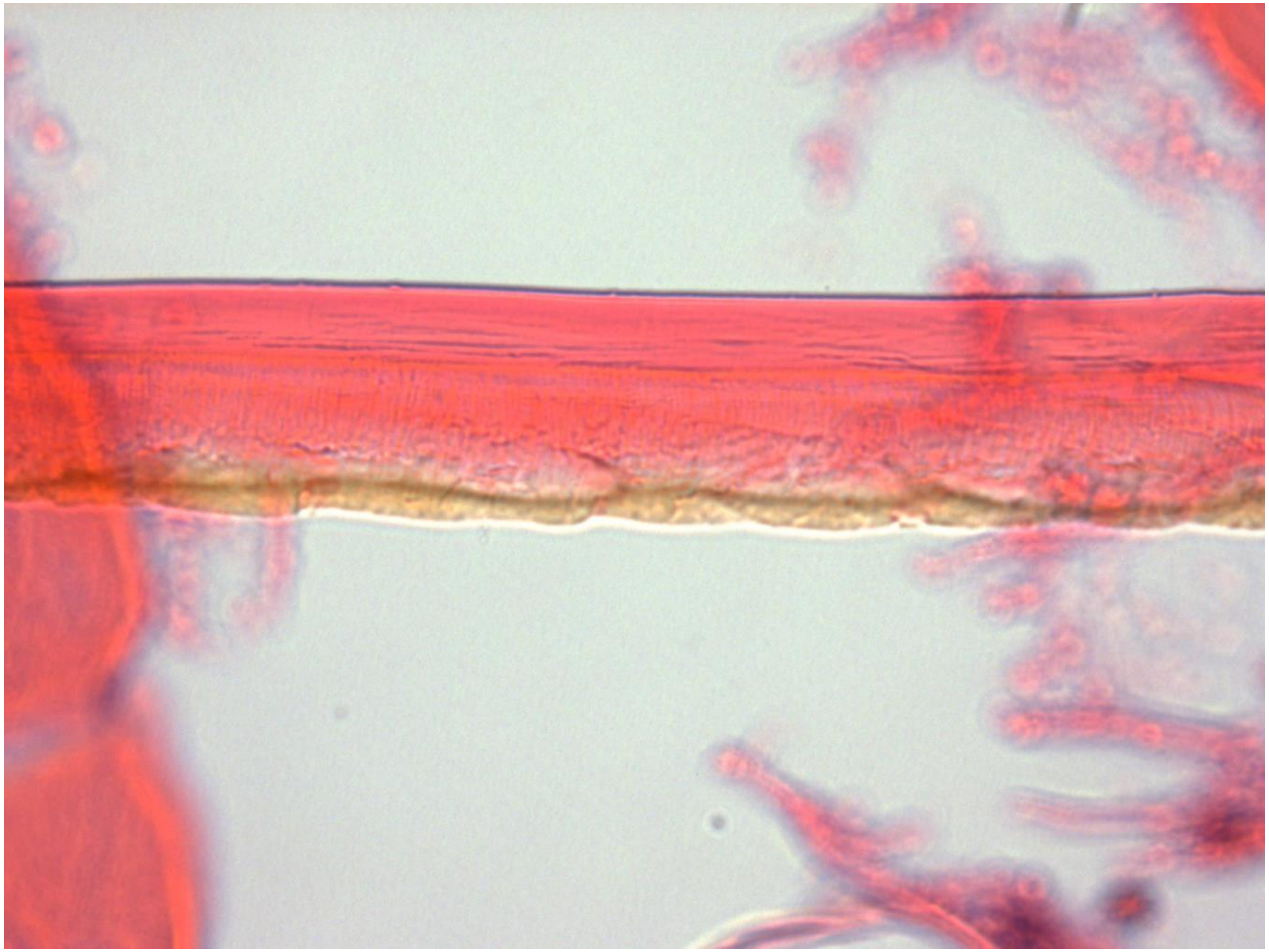

Periplaneta americana #1 Thoracic cuticle 40x magnification

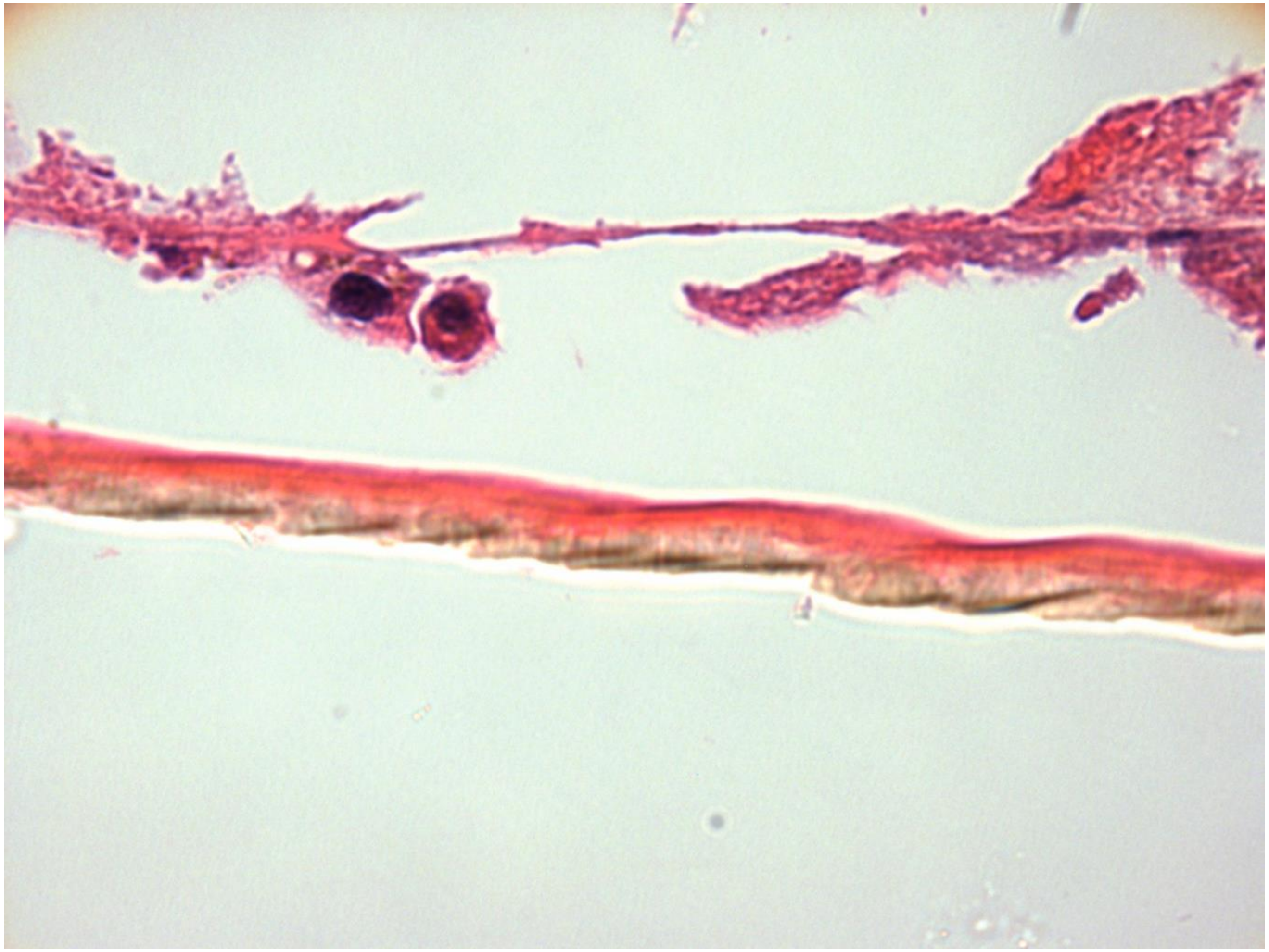

Periplaneta americana #2 **Abdominal cuticle** **40x magnification**

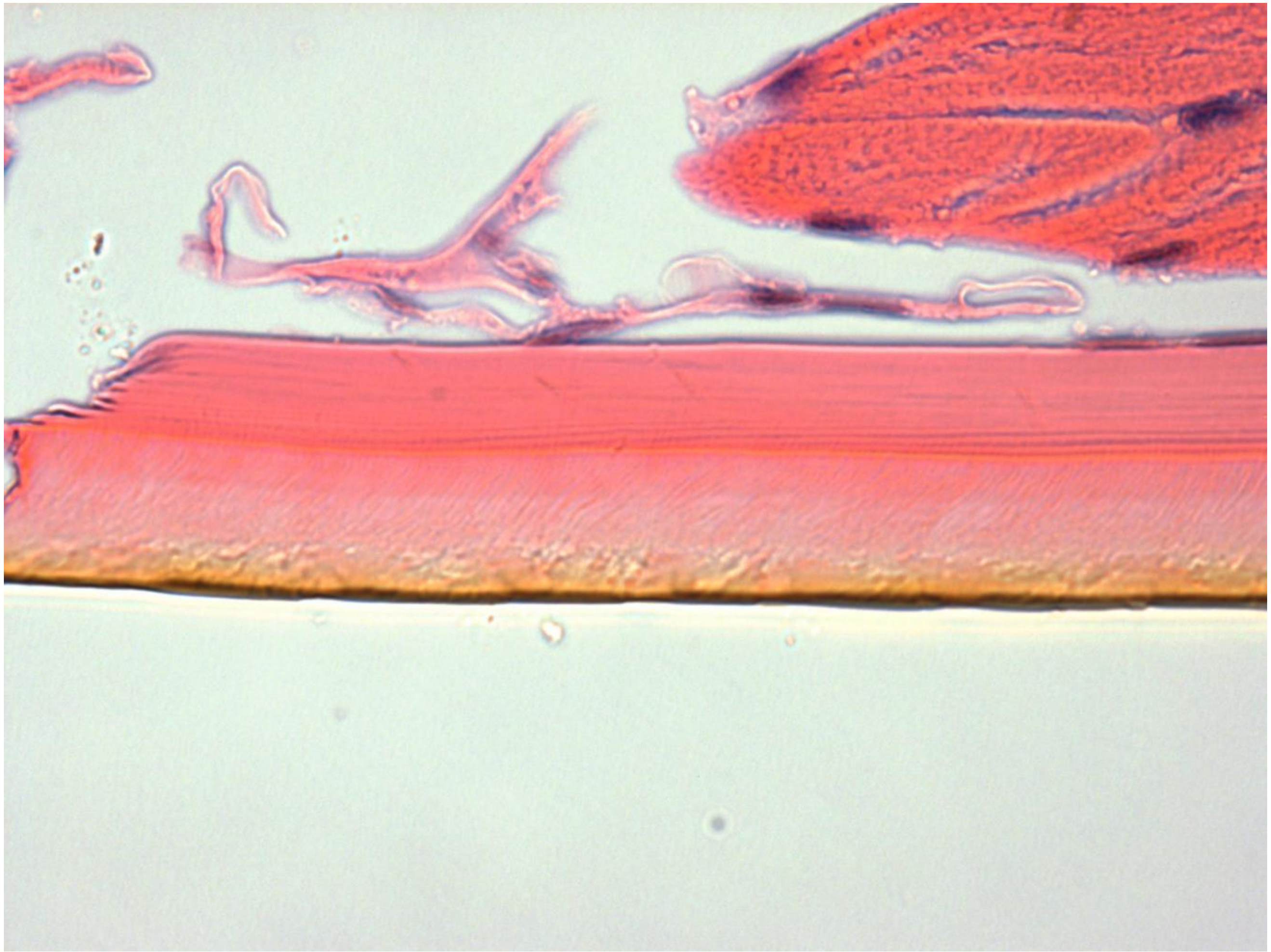

Periplaneta americana **#2** **Thoracic cuticle** **40x magnification**

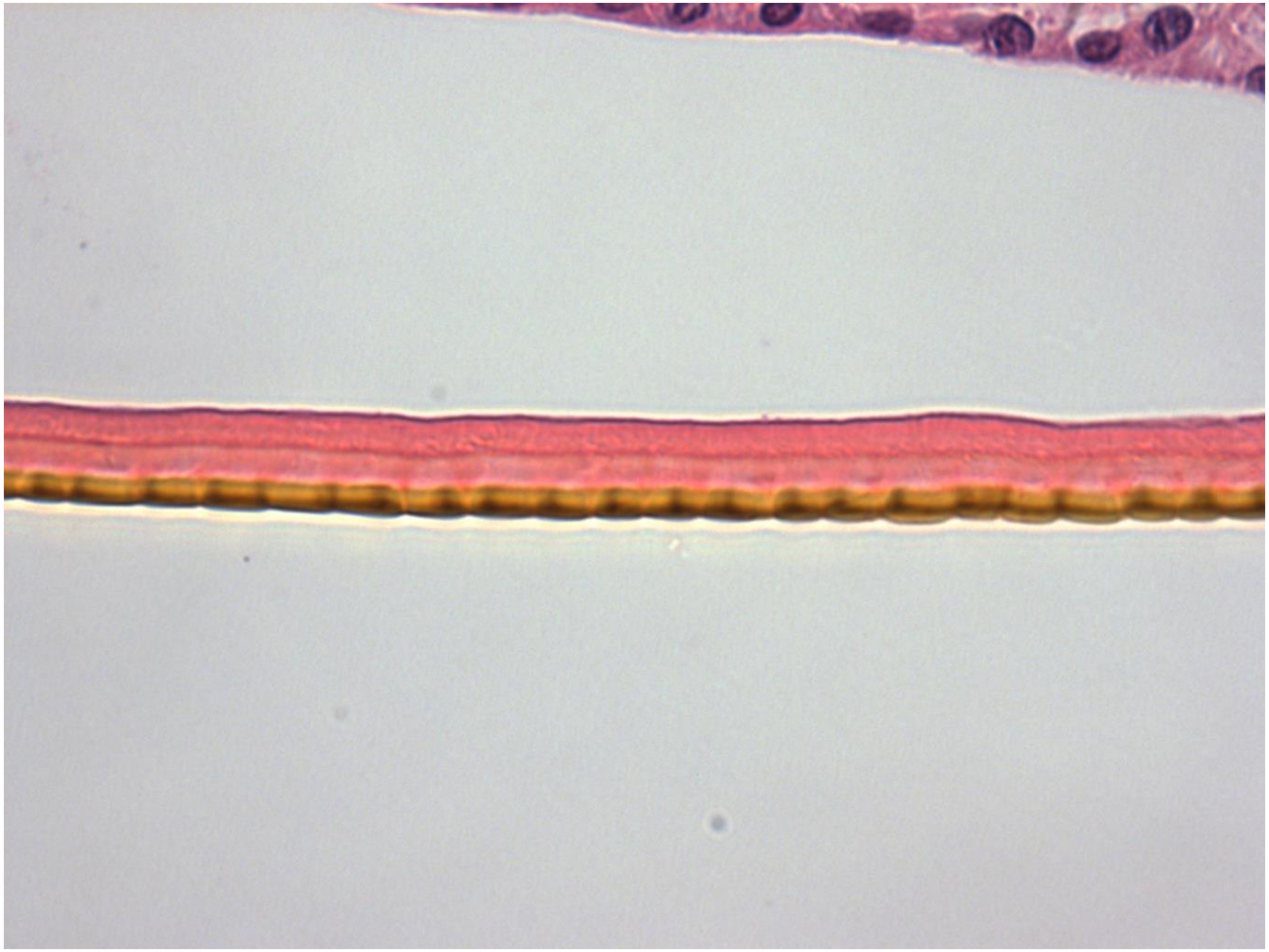

Periplaneta americana #3 **Abdominal cuticle** **40x magnification**

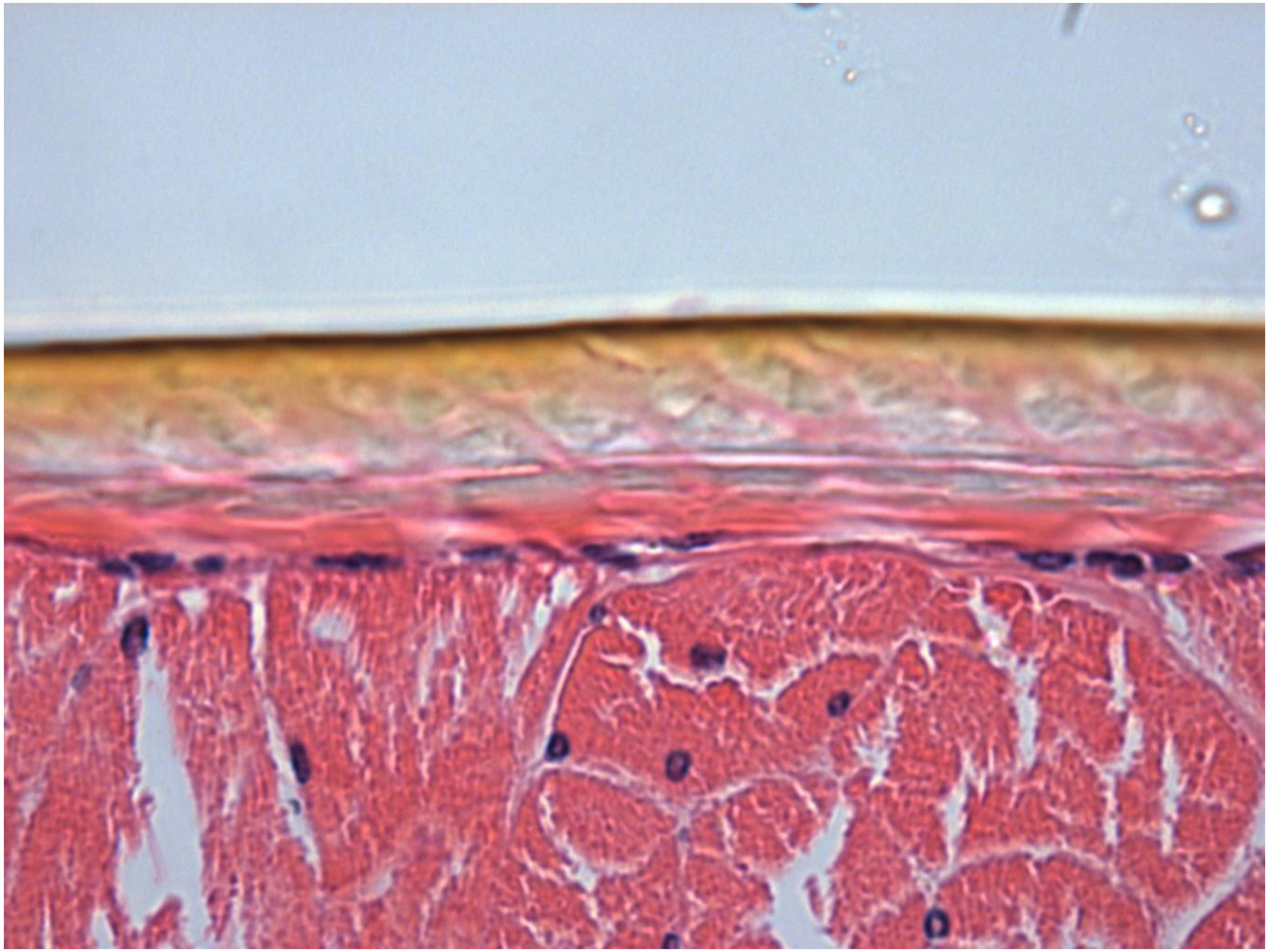

Periplaneta americana #3 **Thoracic cuticle** **40x magnification**

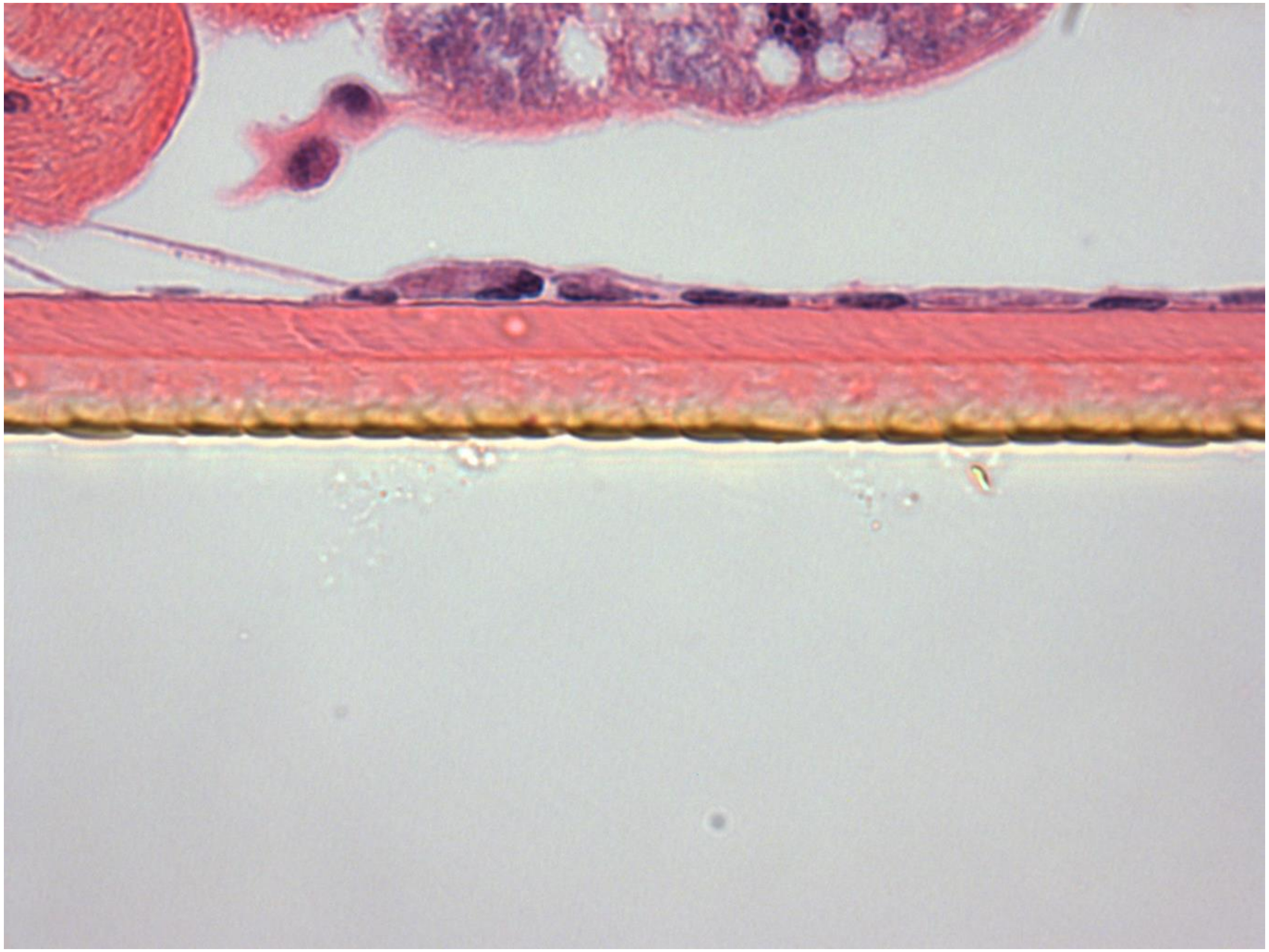

Periplaneta americana #4 **Abdominal cuticle** **40x magnification**

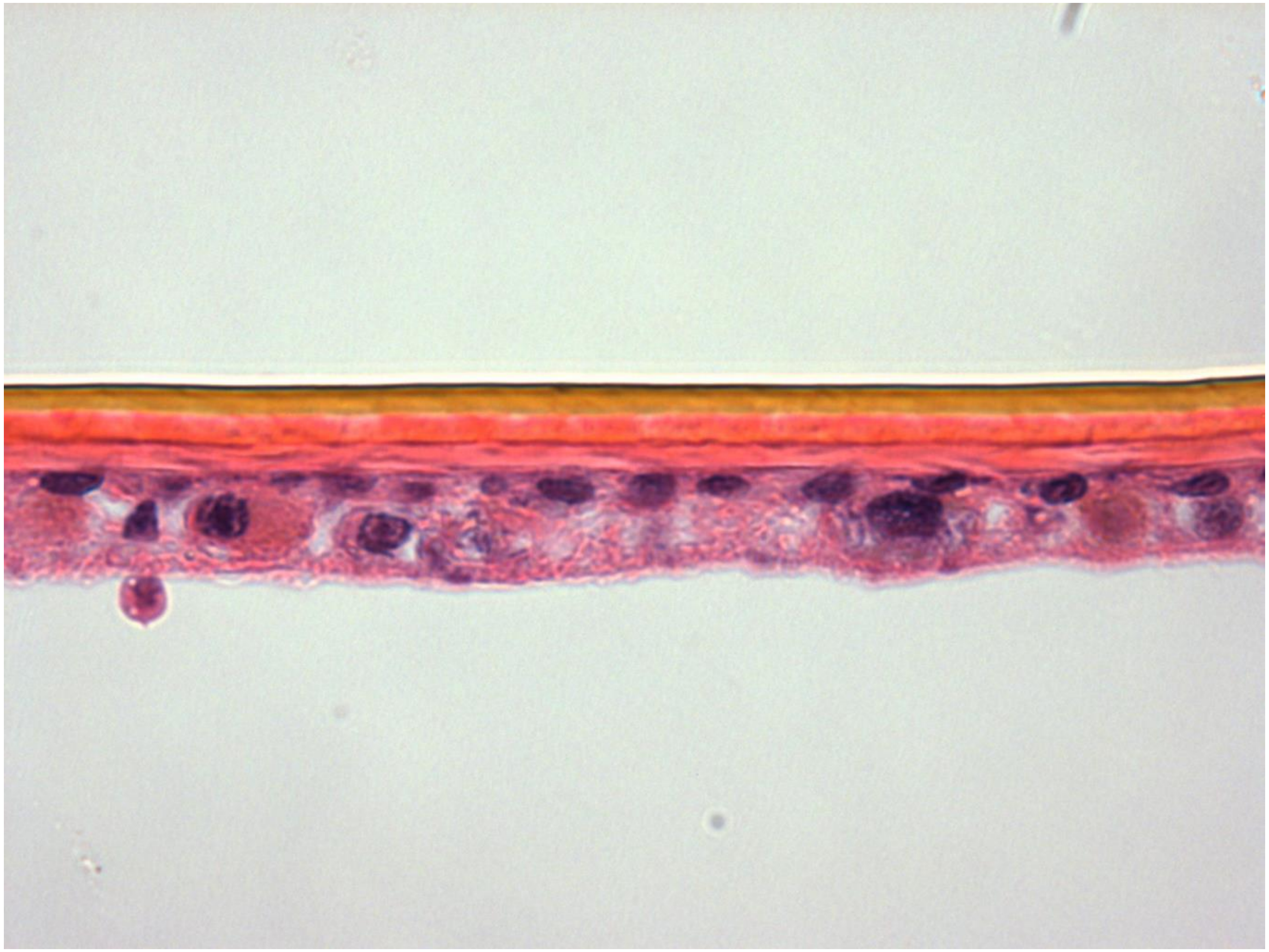

Periplaneta americana #4 Thoracic cuticle 40x magnification

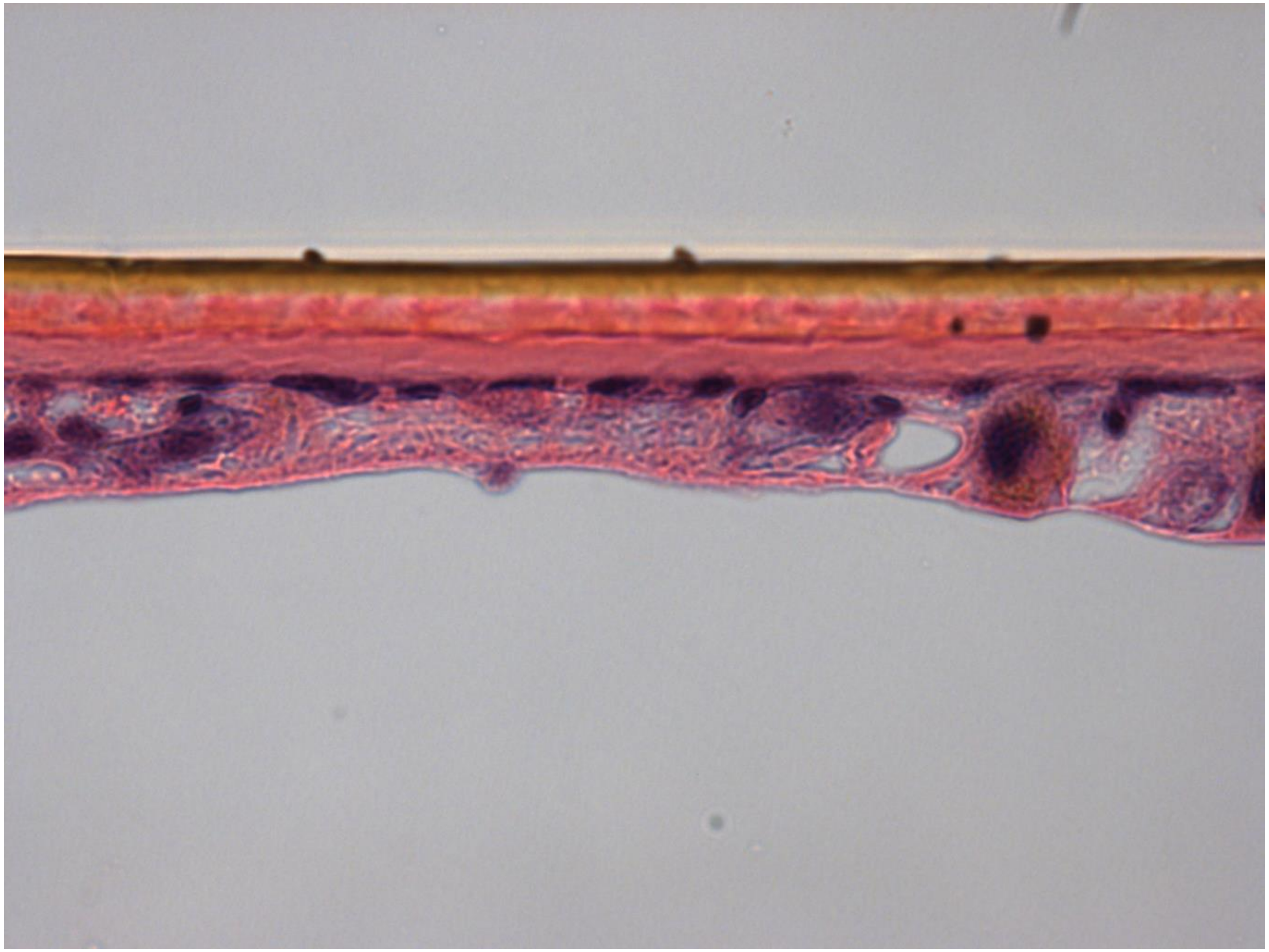

Periplaneta americana #5 **Abdominal cuticle** **40x magnification**

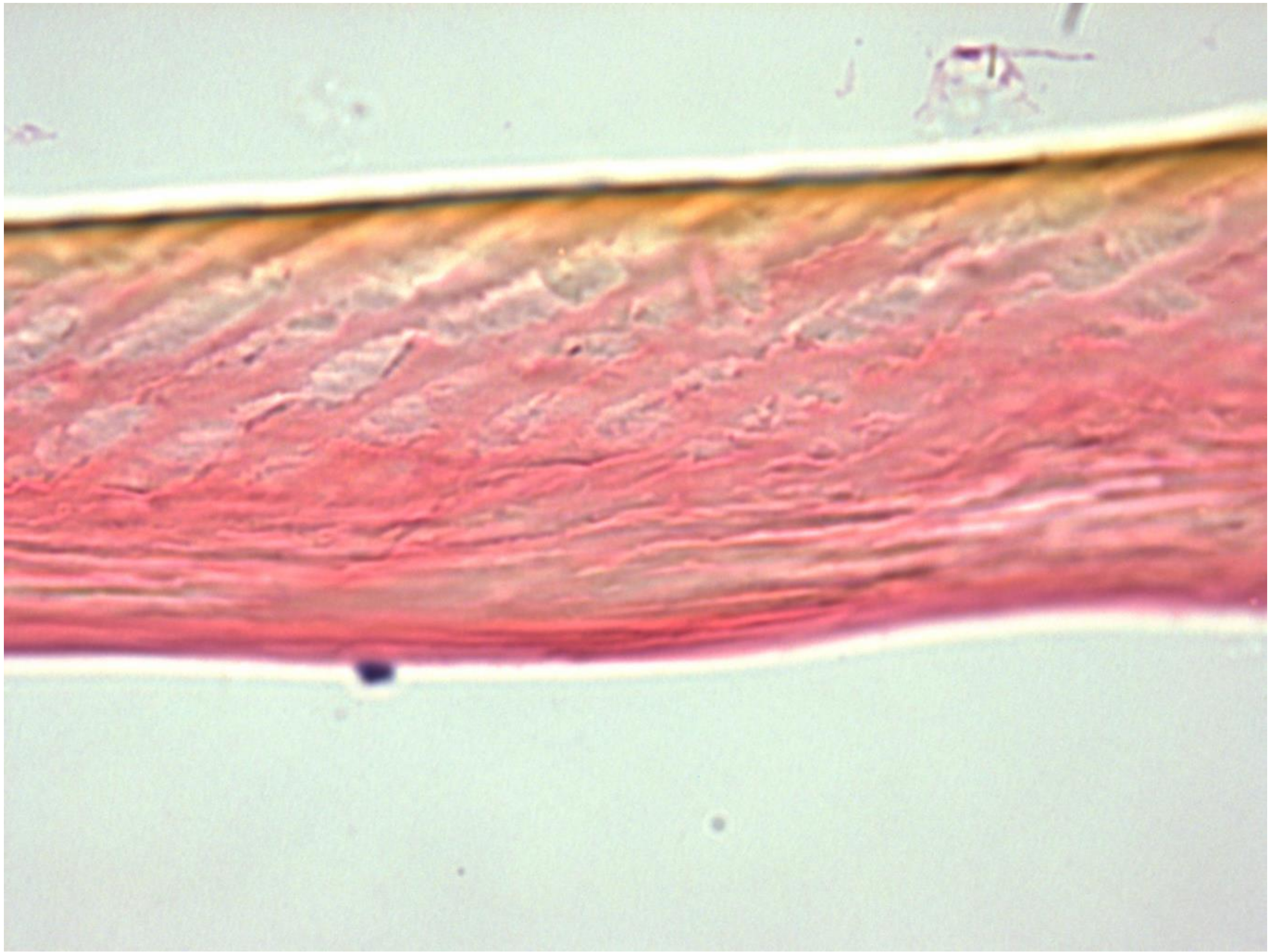

Periplaneta americana #5 **Thoracic cuticle** **40x magnification**

	A	B	C	D	E	F	G	H	I
1	***Periplaneta americana***								
2									
3		40x	**Thickness**	**ABDOMEN**	**PA#1_abd_40x**	**PA#2_abd_40x**	**PA#3_abd_40x**	**PA#4_abd_40x**	**PA#5_abd_40x**
4	Distance in pixels	1624	**(microns)**	(1) Cuticle	19.66	11.03	13.79	18.97	16.38
5	Known Distance	140 microns		(2) Procuticle	18.1	9.14	11.9	16.9	14.66
6		11.6 pixels/micron		(3) Endocuticle	11.72	5	5.17	7.76	6.55
7				Exocuticle	6.38	4.14	6.73	9.14	8.11
8				Epicuticle	1.56	1.89	1.89	2.07	1.72
9									
10		20x		**THORAX**	**PA#1_thor_40x**	**PA#2_thor_40x**	**PA#3_thor_40x**	**PA#4_thor_40x**	**PA#5_thor_40x**
11	Distance in pixels	1682		(1) Cuticle	33.1	36.9	30.86	11.9	66.38
12	Known Distance	290 microns		(2) Procuticle	28.62	35	28.97	11.03	64.83
13		5.8 pixels/micron		(3) Endocuticle	12.93	15.34	12.41	4.14	25.17
14				Exocuticle	15.69	19.66	16.56	6.89	39.66
15				Epicuticle	4.48	1.9	1.89	0.87	1.55
16		10x							
17	Distance in pixels	1798	**Percentages**	**ABDOMEN**	**PA#1_abd_40x**	**PA#2_abd_40x**	**PA#3_abd_40x**	**PA#4_abd_40x**	**PA#5_abd_40x**
18	Known Distance	620 microns		Epicuticle	7.9	17.1	13.7	10.9	10.5
19		2.9 pixels/micron		Exocuticle	32.5	37.5	48.8	48.2	49.5
20				Endocuticle	59.6	45.3	37.5	40.9	40.0
21					100.0	100.0	100.0	100.0	100.0
22				Procuticle	92.1	82.9	86.3	89.1	89.5
23									
24				**THORAX**	**PA#1_thor_40x**	**PA#2_thor_40X**	**PA#3_thor_40x**	**PA#4_thor_40x**	**PA#5_thor_40x**
25				Epicuticle	13.5	5.1	6.1	7.3	2.3
26				Exocuticle	47.4	53.3	53.7	57.9	59.7
27				Endocuticle	39.1	41.6	40.2	34.8	37.9
28					100.0	100.0	100.0	100.0	100.0
29				Procuticle	86.5	94.9	93.9	92.7	97.7
